# Comparative Analysis of PM_2.5_-Bound Polycyclic Aromatic Hydrocarbons (PAHs), Nitro-PAHs (NPAHs), and Water-Soluble Inorganic Ions (WSIIs) at Two Background Sites in Japan

**DOI:** 10.3390/ijerph17218224

**Published:** 2020-11-06

**Authors:** Lu Yang, Lulu Zhang, Hao Zhang, Quanyu Zhou, Xuan Zhang, Wanli Xing, Akinori Takami, Kei Sato, Atsushi Shimizu, Ayako Yoshino, Naoki Kaneyasu, Atsushi Matsuki, Kazuichi Hayakawa, Akira Toriba, Ning Tang

**Affiliations:** 1 Graduate School of Medical Sciences, Kanazawa University, Kakuma-machi, Kanazawa 920-1192, Japan; veronicayl@stu.kanazawa-u.ac.jp (L.Y.); zhang-lulu@se.kanazawa-u.ac.jp (L.Z.); zhanghao@stu.kanazawa-u.ac.jp (H.Z.); zhouquanyu1995@gmail.com (Q.Z.); zhangxuan@stu.kanazawa-u.ac.jp (X.Z.); xingwanli@stu.kanazawa-u.ac.jp (W.X.); 2 National Institute for Environmental Studies, 16-2 Onogawa, Tsukuba, Ibaraki 305-8506, Japan; takamia@nies.go.jp (A.T.); kei@nies.go.jp (K.S.); shimizua@nies.go.jp (A.S.); yoshino.ayako@nies.go.jp (A.Y.); 3 National Institute of Advanced Industrial Science and Technology, 16-1 Onogawa, Tsukuba, Ibaraki 305-8569, Japan; kane.n@aist.go.jp; 4 Institute of Nature and Environmental Technology, Kanazawa University, Kakuma-machi, Kanazawa 920-1192, Japan; matsuki@staff.kanazawa-u.ac.jp (A.M.); hayakawa@p.kanazawa-u.ac.jp (K.H.); 5 Institute of Medical, Pharmaceutical and Health Sciences, Kanazawa University, Kakuma-machi, Kanazawa 920-1192, Japan; toriba@p.kanazawa-u.ac.jp

**Keywords:** PM_2.5_, polycyclic aromatic hydrocarbons, nitro-polycyclic aromatic hydrocarbons, water-soluble inorganic ions, background sites, long-range transport

## Abstract

Daily PM_2.5_ (particulate matter with aerodynamic diameter ≤2.5 μm) samples were simultaneously collected at two background sites (Wajima Air Monitoring Station (WAMS) and Fukue-Jima Atmosphere and Aerosol Monitoring Station (FAMS)) in Japan in the East Asian winter and summer monsoon periods of 2017 and 2019, to compare the characteristics of air pollutants among different regions and to determine the possible variation during the long-range transport process. Polycyclic aromatic hydrocarbons (PAHs), nitro-PAHs (NPAHs), and water-soluble inorganic ions (WSIIs) were analyzed. Despite the PM_2.5_ concentrations at FAMS (8.90–78.5 µg/m^3^) being higher than those at WAMS (2.33–21.2 µg/m^3^) in the winter monsoon period, the average concentrations of ∑PAHs, ∑NPAHs, and ∑WSIIs were similar between the two sites. Diagnostic ratios indicated PAHs mainly originated from traffic emissions and mostly aged, whereas NPAHs were mostly secondarily formed during long-range transport. WSIIs at WAMS were mainly formed via the combustion process and secondary reactions, whereas those at FAMS mainly originated from sea salt and dust. Backward trajectories revealed the air masses could not only come from Asian continental coastal regions but also distant landlocked areas in the winter monsoon period, whereas most came from the ocean in the summer monsoon period. These findings can provide basic data for the establishment of prediction models of transboundary air pollutants in East Asia.

## 1. Introduction

Air pollution is an extremely serious problem worldwide [1,2]. Among air pollutants, PM_2.5_ (particulate matter with aerodynamic diameter ≤2.5 μm) has received extensive attention because a high PM_2.5_ concentration can affect the air quality, climate change, and human health [3,4,5,6,7]. PM_2.5_ consists of a variety of organic and inorganic compounds [8,9,10]. In terms of the organic species, polycyclic aromatic hydrocarbons (PAHs) and nitro-PAHs (NPAHs) are both well-known environmental pollutants due to their carcinogenicity and mutagenicity [11,12,13], although they account for only a small part of PM_2.5_ [14,15]. Regarding the inorganic species, water-soluble inorganic ions (WSIIs) are some of the dominant chemical components of PM_2.5_ that can affect the size, composition, and lifetime of particles owing to their hygroscopic nature [16], thus imposing a large negative effect on the visibility, and they play a key role in the formation of severe pollution events such as haze [17]. Moreover, these organic and inorganic species can be emitted by a wide range of sources [18], and their chemical characteristics continuously vary depending on many factors, such as meteorological conditions and residence time in the atmosphere [19].

Among Eastern Asian countries, Japan is an island country, and it is located on the leeward side of the East Asian monsoon. Due to its specific location, the PM_2.5_ pollution in Japan is not only domestically produced but is also produced on the Asian continent and reaches Japan via long-range transport [20,21,22,23]. Two background sites on the Noto Peninsula (Kanazawa University Wajima Air Monitoring Station; KUWAMS (also called WAMS)) and Fukue Island (Fukue-Jima Atmosphere and Aerosol Monitoring Station; FAMS) in Japan were established to observe atmospheric pollutants that long-range transported from the Asian continent in 2004 and 2002, respectively [24,25,26,27,28,29]. However, observations were only independently performed at each study site, leading to a lack of comprehensive information between these regions. For example, the differences in pressure between the two regions, air pollutant origin sources, and transport patterns can result in differences in air pollutant concentrations and secondary reaction products, leading to many uncertainties in the development of an East Asian observation model.

In this study, we simultaneously collected daily PM_2.5_ samples at WAMS and FAMS in 2017 of April (Period 1, the East Asian winter monsoon period, which means that the cold and dry air mass mostly comes from the Asian continent in the cold period due to the Siberian High [26]) and 2019 of June (Period 2, the East Asian summer monsoon period, which means that the warm and wet air mass mostly comes from the Pacific Ocean in the warm period due to the North Pacific High [26]). Nine PM-bound PAHs, three PM-bound NPAHs, and nine WSIIs in PM_2.5_ were analyzed and compared. The objectives were to further compare the characteristics of air pollutants between WAMS and FAMS in the same period and to determine the possible variation generated during the long-range transport process on the basis of meteorological conditions, PAH, and NPAH diagnostic ratios, backward trajectories, WSII acid–base balance, and potential sources.

## 2. Materials and Methods

### 2.1. PM_2.5_ Sampling

**Figure 1:**
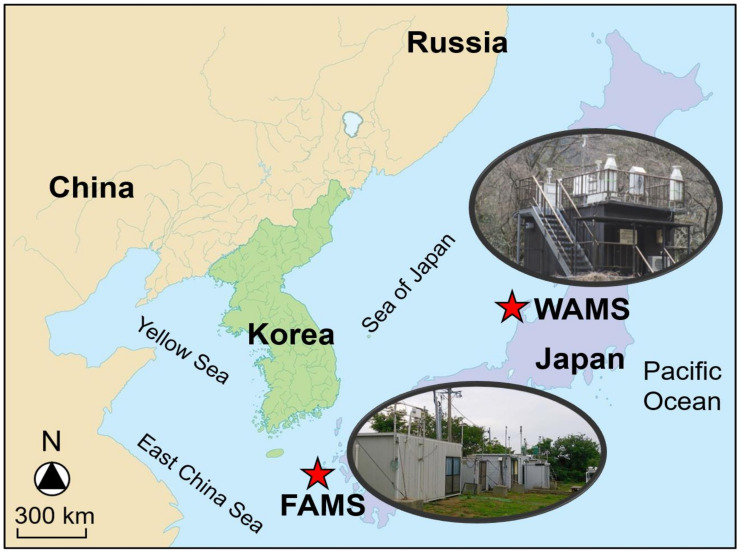
Location of Wajima Air Monitoring Station (WAMS; 37º21′05″ N, 136º47′33″ E) and Fukue-Jima Atmosphere and Aerosol Monitoring Station (FAMS; 32º45′07″ N, 128º40′59″ E), Japan.

As shown in Figure 1, WAMS (Nisifuta-machi, Wajima City, Ishikawa Prefecture, Japan) is located 2.1 km south of the coast in the Sea of Japan at an elevation of approximately 60 m. FAMS (Miirakumachi, Gotō City, Nagasaki Prefecture, Japan) is situated approximately 3 km east of the coast in the East China Sea at an elevation of approximately 30 m. These two sites are both far away from nearby anthropogenic pollution sources.

PM_2.5_ sampling was simultaneously performed at WAMS and FAMS using high-volume air samplers (HV-1000F, Sibata Scientific Technology Ltd., Saitama, Japan) at a flow rate of 1000 L/min, equipped with quartz fiber filters (2500QAT-UP, Pall Co., Port Washington, NY, USA). Filters were changed every 24 h in Period 1 from 10 to 20 April 2017 and in Period 2 from 25 to 29 June 2019. Details on the treatment method of the sampling filters are provided in Text S1 (Supplementary Materials).

### 2.2. PAH, NPAH, and WSII Analysis

The PM_2.5_ samples were twice subjected to ultrasonic extraction after the addition of internal standards (pyrene-*d*_10_ (Pyr-*d*_10_), benzo[*a*]pyrene-*d*_12_ (BaP-*d*_12_), and 2-fluoro-7-nitrofluorene (FNF)), thereafter washed with NaOH (5% *w*/*v*), H_2_SO_4_ (20% *v*/*v*) and distilled water successively, and then concentrated before high-performance liquid chromatography (HPLC, Shimadzu Inc. Kyoto, Japan) was conducted with a fluorescence detection system to detect nine PM-bound PAHs (fluoranthene (FR), Pyr, benz[*a*]anthracene (BaA), chrysene (Chr), benzo[*b*]fluoranthene (BbF), benzo[*k*]fluoranthene (BkF), BaP, benzo[*ghi*]perylene (BgPe), and indeno[1,2,3-*cd*]pyrene (IDP)) and three PM-bound NPAHs (1-nitropyrene (1-NP), 2-nitropyrene (2-NP), and 2-nitrofluoranthene (2-NFR)) (Table S1, Supplementary Materials) [30]. The PM_2.5_ samples were also subjected to ultrasonic extraction by ultrapure water before ion chromatography (883 Basic IC plus, Metrohm, Herisau, Switzerland) was performed to detect nine WSIIs (sodium (Na^+^), ammonium (NH_4_^+^), potassium (K^+^), calcium (Ca^2+^), magnesium (Mg^2+^), chloride (Cl^−^), sulfate (SO_4_^2-^), nitrate (NO_3_^−^), bromine (Br^−^)) (Table S1, Supplementary Materials) [31]. In this study, the calibration curves of PAHs, NPAHs, and WSIIs standard solution all had good linearity (*r* > 0.998). The recoveries of PAHs and NPAHs internal standards ranged from 80% to 95%. The detailed sample pretreatment and quality control and quality assurance processes are described in Texts S2 and S3 (Supplementary Materials), respectively.

In this study, the chemicals for the target analysis species, including US EPA 610 PAH mix were purchased from Supelco Park; 1-NP, 2-NP, and FNF were acquired from the Aldrich Chemical Company, while 2-NFR was purchased from Chiron AS. Pyr-*d*_10_, BaP-*d*_12,_ and WSII standard solutions were obtained from Wako Pure Chemicals.

### 2.3. Data Analysis

The daily meteorological conditions including temperature, precipitation, relative humidity, wind speed, and direction at WAMS and FAMS in Periods 1 and 2 were retrieved from the Japan Meteorological Agency (http://www.jma.go.jp/jma/menu/menureport.html).

Backward trajectories were calculated every hour at a sampling point height of 1000 m above ground level, and a tracking time of 72 h was adopted for each trajectory in Periods 1 and 2, using the United States (US) National Oceanic and Atmospheric Association’s Hybrid Single-Particle Lagrangian Integrated Trajectory (HYSPLIT4) model (WINDOWS-based). Frequency analysis of all backward trajectories was performed according to the spatial distribution characteristics of the backward trajectories in Periods 1 and 2 at WAMS and FAMS.

Statistical analysis of the data was performed using the IBM SPSS 25.0 software package (IBM, Armonk, NY, USA). Spearman correlation analysis was conducted to determine the correlations between the target species and relative meteorological conditions. At *p* < 0.05, the results were considered significant. Principle component analysis (PCA) was applied to identify the potential PCA and WSII emission sources.

## 3. Results

### 3.1. Concentrations

**Figure 2:**
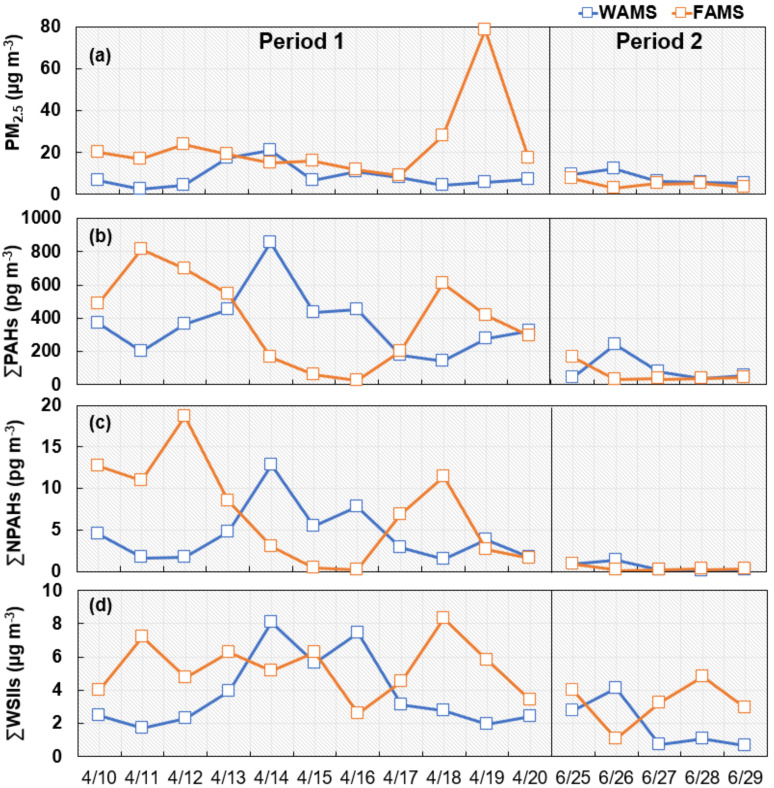
Concentrations of particulate matter with aerodynamic diameter ≤2.5 μm (PM_2.5_) (**a**), total polyaromatic hydrocarbons (∑PAHs) (**b**), total nitro-PAHs (∑NPAHs) (**c**), and total water-soluble inorganic ions (∑WSIIs) (**d**) at WAMS and FAMS in the East Asian winter monsoon period (Period 1, 10–20 April 2017) and summer monsoon period (Period 2, 25–29 June 2019).

As shown in Figure 2a, in Period 1, the daily average PM_2.5_ concentration at WAMS was 8.62 µg/m^3^ (range: 2.33–21.2 µg/m^3^), mostly lower than the 23.2 µg/m^3^ (range: 8.90–78.5 µg/m^3^) observed at FAMS. The long-range transported Asian dust observed at FAMS from 18 to 19 April (*the Japan Meteorological Agency*) led to the PM_2.5_ concentration at the site increasing from 27.9 to 78.5 µg/m^3^, which was much higher than that on the other days. In Period 2, the daily average PM_2.5_ concentration at WAMS was 7.88 µg/m^3^ (range: 5.47–12.4 µg/m^3^), while that at FAMS was 4.96 µg/m^3^ (range: 2.79–7.51 µg/m^3^). Except for the concentration on 26 June, the daily concentration difference was small.

Figure 2b reveals that, in Period 1, the ∑PAHs concentrations ranged from 144 to 856 pg/m^3^ at WAMS and from 27.6 to 811 pg/m^3^ at FAMS. Compared to our previous studies conducted in April 2009 (WAMS: 583 pg/m^3^; FAMS: 582 pg/m^3^) and April 2010 (WAMS: 565 pg/m^3^; FAMS: 625 pg/m^3^) [27,32], the average concentrations at WAMS (368 pg/m^3^) and FAMS (392 pg/m^3^) both decreased. Although the PM_2.5_ concentration was the highest at FAMS on 19 April (Figure 2a) due to Asian dust, the PAH concentration was not the highest (Figure 2b), consistent with a previous study whereby not every Asian dust event exhibits a high PAH concentration [33]. In Period 2, the daily average ∑PAHs concentration at WAMS was 88.8 pg/m^3^ (range: 34.2–241 pg/m^3^), while that at FAMS was 61.9 pg/m^3^ (range: 28.6–167 pg/m^3^). Although the average concentrations at WAMS and FAMS were similar in both Periods 1 and 2, the daily concentration significantly varied, which exhibited the opposite trend at these two sites. The daily concentration differences ranged from 25.0 to 693 pg/m^3^ in Period 1 and from 5.26 to 213 pg/m^3^ in Period 2.

As shown in Figure 2c, in Period 1, the daily ∑NPAHs concentration at WAMS was 4.44 pg/m^3^ (range: 1.46–12.9 pg/m^3^), while that at FAMS was 7.01 pg/m^3^ (range: 0.49–18.7 pg/m^3^). Except for the concentration on 20 April at WAMS and from 10 to 12 April at FAMS, the daily concentration at the two sites revealed a similar trend to that of ∑PAHs, but the ∑NPAHs concentration differences were much smaller than those of ∑PAHs (from 0.20 to 17.0 pg/m^3^). In Period 2, the ∑NPAHs concentrations at WAMS and FAMS were mostly lower than 1 pg/m^3^ except for those on 26 June at WAMS (1.38 pg/m^3^).

Figure 2d shows that, in Period 1, the daily average ∑WSIIs concentration at WAMS was 3.81 µg/m^3^ (range: 1.72–8.06 µg/m^3^), while that at FAMS was 5.31 µg/m^3^ (range: 2.60–8.34 µg/m^3^). Although the daily concentration exhibited a similar change trend to that of PM_2.5_ at WAMS, a different change trend was observed at FAMS. The daily concentration differences ranged from 0.63 to 5.56 µg/m^3^. In Period 2, the daily average ∑WSIIs concentration at WAMS was 1.87 µg/m^3^ (range: 0.70–4.10 µg/m^3^), while that at FAMS was 3.22 µg/m^3^ (range: 1.08–4.81 µg/m^3^). In contrast to PM_2.5_, ∑PAHs, and ∑NPAHs, the ∑WSIIs daily concentration variation clearly differed between these two sites, especially from 27 to 29 June.

### 3.2. Compositions

As shown in Figure S1 (Supplementary Materials), in Period 1, the dominant PAHs were FR, Pyr, BbF, and BgPe at both WAMS and FAMS. The daily percentage of four-ring (FR + Pyr + BaA + Chr) (54%–64%), five-ring (BbF + BkF + BaP) (19%–24%), and six-ring (BgPe + IDP) (17%–22%) PAHs at WAMS (Figure S1a, Supplementary Materials) did not vary greatly, while the daily percentage of each individual PAH at FAMS (Figure S1b, Supplementary Materials) clearly differed. In Period 2, the percentage of each PAH at both WAMS (Figure S1c, Supplementary Materials) and FAMS (Figure S1d, Supplementary Materials) greatly varied on different days. Tables S2 and S3 and Figure S2 (Supplementary Materials) indicate that the daily concentration of 2-NFR accounted for a large proportion of ∑NPAHs on most days at both WAMS and FAMS in Periods 1 and 2. However, the concentration of 1-NP in most samples was below the limit of detection (LOD) at both WAMS and FAMS in Period 2 (Tables S2 and S3, Supplementary Materials).

As shown in Figure S3 (Supplementary Materials), the SO_4_^2−^ concentrations contributed much to ∑WSIIs at both WAMS (Figure S3a, Supplementary Materials) and FAMS (Figure S3b, Supplementary Materials) in Periods 1 and 2. In Period 1, SO_4_^2−^, NH_4_^+^, and NO_3_^−^ were the three dominant species of ∑WSIIs on most days at both WAMS and FAMS, and these three species accounted for at least 77.9% and 63.8% of ∑WSIIs at WAMS and FAMS, respectively. In Period 2, only SO_4_^2−^, NH_4_^+^, and NO_3_^−^ were mostly detected at WAMS (Table S2, Supplementary Materials), and only SO_4_^2−^, NH_4_^+^, and Na^+^ were mostly detected at FAMS (Table S3, Supplementary Materials). On the other hand, Figure S3 (Supplementary Materials) shows that the daily percentage of ∑WSIIs in PM_2.5_ ranged from 23.0% to 85.3% at WAMS in Period 1, it ranged from 7.4% to 50.7% at FAMS in Period 1, it ranged from 11.1% to 33.1% at WAMS in Period 2, and it ranged from 38.5% to 87.3% at FAMS in Period 2. This result indicated that more species occurred in PM_2.5_ at WAMS than at FAMS in Period 2, but the opposite was true in Period 1.

## 4. Discussion

### 4.1. Meteorological Conditions

**Figure 3:**
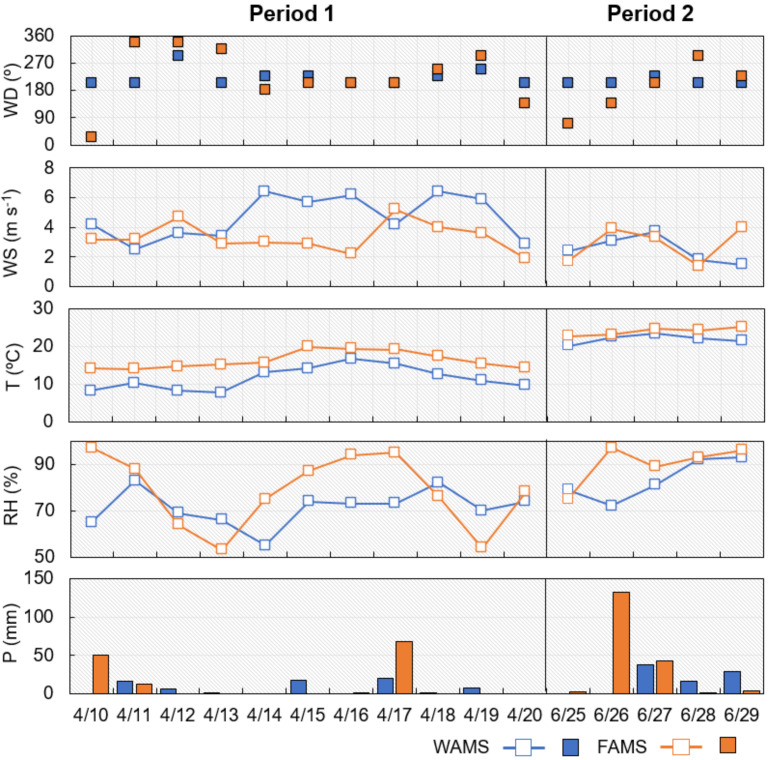
Meteorological conditions at WAMS and FAMS in the East Asian winter monsoon period (Period 1, 10–20 April 2017) and summer monsoon period (Period 2, 25–29 June 2019). P, RH, T, WS, and WD are precipitation, relative humidity, temperature, wind speed, and wind direction, respectively.

Meteorological conditions play a key role in affecting air pollutants in terms of their characteristics, such as phase partitioning, accumulation, diffusion, and removal [34]. Figure 3 reveals the diurnal variations in the meteorological conditions in the two sampling periods. The ambient temperature at WAMS was slightly lower than that at FAMS in both Periods 1 and 2, but the variation at each site was not notable. Therefore, the concentration variation in PM_2.5_, ∑PAHs, ∑NPAHs, and ∑WSIIs was relatively independent of the temperature in this study (Spearman’s correlation, *p* > 0.05).

Figure 3 shows that the wind speed varied between approximately 2 and 6 m/s at both WAMS and FAMS in Period 1. However, the wind direction was more stable at WAMS than at FAMS, suggesting that the source areas were similar at WAMS and were different at FAMS. Therefore, a lower variation in the daily percentage of each individual PAH was observed at WAMS than at FAMS in Period 1 (Figure S1, Supplementary Materials; Section 3.2). Previous studies reported that the North Pacific High (subtropical anticyclone in the Pacific Ocean with warm and wet air masses) greatly influenced Japan in Period 2 [21,26]. Therefore, meteorological conditions such as precipitation and relative humidity can notably affect the daily percentage variations in each individual PAH at both WAMS and FAMS (Figure S1, Supplementary Materials; Section 3.2). In addition, the lowest concentrations of PM_2.5_, ∑PAHs, ∑NPAHs, and ∑WSIIs were observed at FAMS on 26 June (Figure 2) with a high precipitation (Figure 3).

### 4.2. Diagnostic Ratios

**Figure 4:**
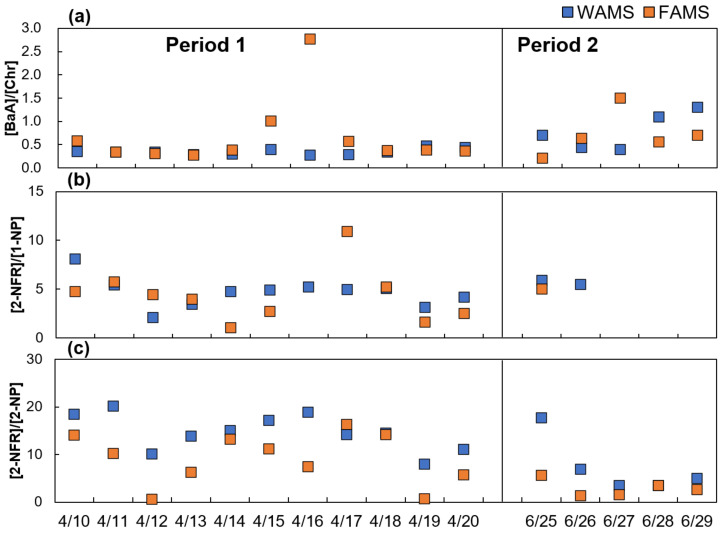
Diagnostic ratios of [BaA]/[Chr] (**a**), [2-NFR]/[1-NP] (**b**) and [2-NFR]/[2-NP] (**c**) at WAMS and FAMS in the East Asian winter monsoon period (Period 1, 10–20 April 2017) and summer monsoon period (Period 2, 25–29 June 2019).

Because WAMS and FAMS are both far away from anthropogenic pollution sources, external sources, influenced by long-and short-range transport processes, are important mechanisms to explain the concentration of pollutants in the atmosphere. The impact of long-range transport is reflected by the aging degree of the air mass. BaA can degrade more easily than Chr in the atmosphere [35]. Hence, the [BaA]/[Chr] ratio (1.0) can be adopted to illustrate whether an air mass is fresh or aged during long-range transport [36]. A higher ratio indicates relatively low photochemical reactions and a major impact on local emission or short-range transport [36]. As shown in Figure 4a, in Period 1, most [BaA]/[Chr] ratios were relatively low at both WAMS and FAMS, representing aged air masses, implying that more PAHs degraded during the long-range transport process. However, the [BaA]/[Chr] ratios were higher than 1.0 at FAMS on 15 and 16 April, which indicates typical local sources or short-range transport [37]. The backward trajectory is examined in detail in Section 4.3. In Period 2, the [BaA]/[Chr] ratios greatly varied at both WAMS and FAMS, indicating alternating influences of short- and long-range transport processes.

Among the three NPAHs, 2-NFR and 2-NP are secondarily formed, and 1-NP is primarily formed in the atmosphere [38,39]. To evaluate the relative contribution of primary emission and secondary formation of NPAHs, the [2-NFR]/[1-NP] ratio was applied. A previous study reported that values less than 5 were typically observed at the sites near primary emission sources [38]. Moreover, a higher ratio of [2-NFR]/[1-NP] can also be associated with the longer exposure time of air masses at the background site due to the photochemical reaction and less anthropogenic emission [38]. As shown in Figure 4b, in Period 1, the [2-NFR]/[1-NP] ratios at FAMS varied more than those at WAMS, but most of the ratios were near or less than 5 at both sites, indicating the importance of primary emission or revealing the relatively short exposure time of air masses. According to the similar results of a previous study conducted at WAMS [40], 2-NFR was possibly secondarily formed on the Asian continent and then transported across a long distance to Japan in a short time or secondarily formed during the long-range transport process. Moreover, it surmises that 1-NP was possibly contained during the long-range transport process, leading to a low ratio of [2-NFR]/[1-NP]. The [2-NFR]/[2-NP] ratio is associated with the OH radical-initiated reaction (a ratio of approximately 10) and the NO_3_ radical-initiated reaction (a ratio of approximately 100) [38]. Figure 4c reveals that the [2-NFR]/[2-NP] ratios at the two sites approached 10, i.e., much lower than 100, in both Periods 1 and 2, indicating that the OH radical-initiated reaction was the main pathway of the secondary 2-NFR and 2-NP detected at both WAMS and FAMS.

### 4.3. Backward Trajectory Analysis

As discussed in Section 4.2, PAHs and NPAHs were mostly transported across a long distance to WAMS and FAMS. The long-range transport of air masses can carry pollutants from the Asian continent or clean air from ocean areas, thus influencing the concentration of pollutants at WAMS and FAMS [21,26,27]. In this study, the frequency analysis results of the backward trajectories at WAMS and FAMS in Periods 1 and 2 are shown in Figure 5 and Figure 6, respectively. The daily frequencies of the backward trajectories at WAMS and FAMS in Periods 1 and 2 are shown in Figures S4–S19 (Supplementary Materials).

**Figure 5:**
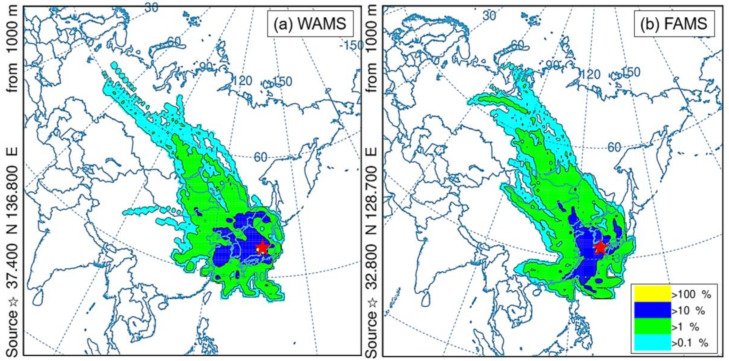
Frequency analysis of backward trajectories at WAMS (**a**) and FAMS (**b**) in the East Asian winter monsoon period (Period 1, 10–20 April 2017). (★) sampling sites at WAMS and FAMS.

**Figure 6:**
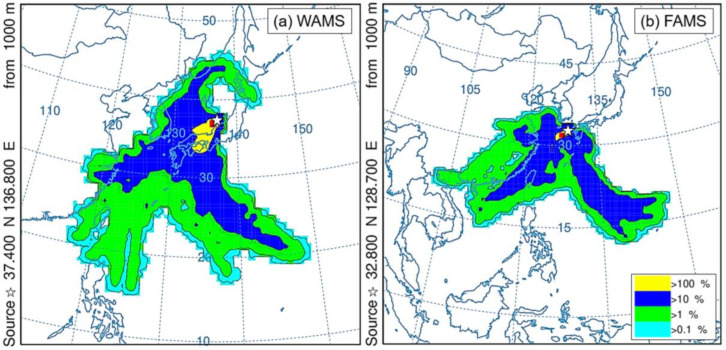
Frequency analysis of backward trajectories at WAMS (**a**) and FAMS (**b**) in the East Asian summer monsoon period (Period 2, 25–29 June 2019). (☆) sampling sites at WAMS and FAMS; (■) the longest air mass residence area.

In Period 1 (Figure 5), the East Asian winter monsoon highly impacted WAMS and FAMS. This indicates that the long-range transported air masses arriving in Japan can come not only from coastal regions in Asian continental countries but also from more distant landlocked areas. However, the data reflect the different main impact areas at WAMS and FAMS. Specifically, at WAMS, a frequency higher than 10% was mainly associated with air masses from northeastern China, Korea, the far eastern area of Russia, the western area of Japan, and the ocean (Figure 5a), while the similar air masses at FAMS primarily stemmed from the Beijing–Tianjin–Hebei region of China, South Korea, Kitakyushu, Japan, and the ocean (Figure 5b). Moreover, the air masses traveling to FAMS dispersed from the Asian continent to the ocean on a daily basis, and the areas with the longest air mass residence times were the Yellow Sea and the East China Sea from 13 to 16 April, whereas the air masses traveling to WAMS still mainly came from the Asian continent (Figures S6–S10, Supplementary Materials). The air mass traveled across the ocean over a longer period and, therefore, the air parcels were cleaner and contained less ∑PAHs and ∑NPAHs, resulting in the concentrations at FAMS continually decreasing from 13 to 16 April (Figure 2b,c). On the other hand, because the air masses did not have to travel across a long distance from the Asian continent to reach FAMS, BaA was less degraded during the short-range transport process, leading to the [BaA]/[Chr] ratio increasing from 13 April, reaching its highest value on 16 April (Figure 4a; Section 4.2). Because 1-NP is the tracer of the traffic emission and there were less anthropogenic sources near the site, fewer air masses traveling across a long distance from the Asian continent to reach FAMS could have led to the decrease in 1-NP concentration, even to a concentration less than the LOD on 16 April (Table S3, Supplementary Materials). Meanwhile, this could have led to the [2-NFR]/[1-NP] ratio increasing (Figure 4b). Moreover, a previous study reported that NO_3_^−^ had a strong seasonal trend of the size distribution due to its higher vapor pressure, which was mostly located in fine particles in autumn and winter and in coarse particles in spring and summer [41]. Furthermore, NO_3_^−^ in PM_2.5_ is mainly produced by the heterogeneous oxidation of NO_x_ emitted from traffic exhaust [41]. Thus, the concentrations of NO_3_^−^ at FAMS were relatively low from 14 to 16 April (Figures S8 to S10, Supplementary Materials) and in Period 2 (Figure 6; next paragraph) due to the fewer air masses traveling across a long distance from the Asian continent and the less anthropogenic sources near the site.

In Period 2 (Figure 6), there was almost no air mass impact observed from the Asian continent on either WAMS or FAMS. At WAMS, frequencies higher than 10% were observed for air masses mainly coming from the west area of Japan and the ocean (Figure 6a), while, at FAMS, they primarily originated from the southern coastal areas of China and the ocean (Figure 6b). The same results are also shown in Figures S15 to S19 (Supplementary Materials), consistent with our previous studies, whereby air masses were strongly influenced by source transport from the ocean and Japan at WAMS and dominated by source transport from the ocean in warmer periods at FAMS [26,27,28,32].

### 4.4. Acid–Base Balance of WSIIs

**Figure 7:**
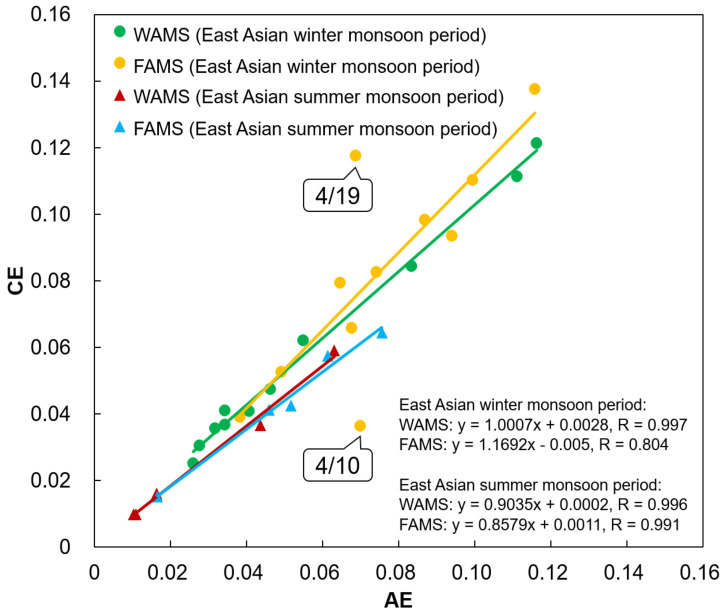
WSII acid–base balance at WAMS and FAMS in the East Asian winter monsoon period (Period 1, 10–20 April 2017) and summer monsoon period (Period 2, 25–29 June 2019).

The cation equivalent (CE) to anion equivalent (AE) ratio is commonly employed to evaluate the acidity of environmental samples [41]. According to the calculation equations of AE and CE provided in Text S4 (Supplementary Materials) [42], the correlations between AE and CE at WAMS and FAMS in Periods 1 and 2 were obtained, as shown in Figure 7. Good relationships occurred between AE and CE at both WAMS and FAMS in Periods 1 and 2, and the results showed that WSIIs at WAMS (Period 1: 1.00; Period 2: 0.90) were more neutral than those at FAMS (Period 1: 1.17; Period 2: 0.86). The CE/AE values at WAMS and FAMS were higher in Period 1 than those in Period 2, indicating that WSIIs in Period 2 were more acidic than those in Period 1. It is likely that alkaline cations such as Ca^2+^ were more frequently collected at WAMS and FAMS in Period 1 (Tables S1 and S2, Supplementary Materials). For example, Figure 7 shows that WSIIs were distinctly alkaline at FAMS on 19 April. Figure S3 (Supplementary Materials) reveals that the percentages of Ca^2+^ and Mg^2+^ notably increased at FAMS from 18 to 19 April, and Spearman correlation analysis indicated a significant positive relation between Ca^2+^ and Mg^2+^ (*r* = 0.80, *p* < 0.01) at FAMS in Period 1, indicating a similar source. The [Ca^2+^]/[Na^+^] ratios of these two days at FAMS were 1.68 and 7.74, and the [Mg^2+^]/[Na^+^] ratios were 0.28 and 0.52, respectively, much higher than the ratios of seawater sources ([Ca^2+^]/[Na^+^]: 0.038; [Mg^2+^]/[Na^+^]: 0.12) [43]. Previous studies demonstrated that Ca^2+^ and Mg^2+^ can occur in crustal material [42,44,45]. The high PM_2.5_ concentration suggested that long-range transport of Asian dust to FAMS occurred from 18 to 19 April (Figure 2a, Section 3.1). The ratios of [BaA]/[Chr] in these two days were much lower than 1.0, also representing aged PAHs due to the long-range transport (Figure 4). The backward trajectories shown in Figures S12 and S13 (Supplementary Materials) reveal that a part of the air masses originated from northern China on 18 April and all the air masses originated from northern China on 19 April, i.e., areas containing the Gobi Desert and the border between China with Mongolia. Therefore, Asian dust containing high concentrations of Ca^2+^ and Mg^2+^ was transported to FAMS on 19 April, leading to more alkaline WSIIs. On the other hand, Figure 2 and Table S3 (Supplementary Materials) show that the concentrations of nine individual PAHs on 18 April were higher than those on 19 April, indicating that PAHs mostly originated from central and southern China on 18 April (Figure S12, Supplementary Materials) and the Asian dust did not contain many PAHs in this event. This further led to less secondary formation of NPAHs due to the decrease in parent PAHs.

Moreover, the CE/AE value on 10 April at FAMS also showed an outlier. As shown in Figure 3, the high precipitation and wind direction on 10 April differed greatly from those on the other days at FAMS, and it is surmised that wet deposition and emission sources jointly affected the acid–base balance of WSIIs.

### 4.5. Potential Sources

Because WAMS and FAMS are both near the sea, parts of WSIIs in PM_2.5_ possibly came from sea salt. In this study, the impact of non-sea salt (nss; SO_4_^2−^, K^+^, and Ca^2+^) in PM_2.5_ was evaluated using the method shown in Text S5 (Supplementary Materials) [41]. It is assumed that Na^+^ in PM_2.5_ only originated from sea salt and the concentrations of nss species could be calculated using the ratios of those species to Na^+^ in seawater. Then, the impact of sea salt source in PM_2.5_ was evaluated using the ratio of nss species concentration to the total concentration of these species in WSIIs. According to the ratios of non-sea salt WSII factions shown in Table S4 (Supplementary Materials), SO_4_^2−^ was considered a secondary compound because the non-sea salt SO_4_^2−^ (nss-SO_4_^2−^) accounted for more than 95% of SO_4_^2−^ at both WAMS and FAMS. K^+^ was mostly the nss-K^+^ species from biomass combustion [46]. Although most [nss-Ca^2+^]/[Ca^2+^] ratios provided in Table S4 (Supplementary Materials) accounting for more than 80% of Ca^2+^ at both WAMS and FAMS were considered as a dust source, the decrease in [nss-Ca^2+^]/[Ca^2+^] from 18 to 20 April at WAMS suggested some impact of sea salt [41]. Moreover, it could not be evaluated on some days at FAMS because the concentrations of Ca^2+^ were below the LOD.

PCA is a useful multivariate statistical tool to identify the potential emission sources of air pollutants [47]. In this study, PCA was applied to PAHs, NPAHs, and WSIIs, and the loadings of each species at WAMS and FAMS in the Asian monsoon period are listed in Table 1. Four factors could explain 96.3% of the total variance at WAMS, and five factors could explain 96.8% of the total variance at FAMS. Regarding WAMS, PC 1 (75.1%) had high loadings of all PAHs species, 2-NP, 1-NP, K^+^, and NO_3_^−^, suggesting the co-effects of combustion emission and secondary reaction [48]. PC 2 (11.3%) had high loadings of NH_4_^+^ and SO_4_^2−^, suggesting secondary reactions [41]. Moreover, 2-NFR had relatively high loading in both PC 1 (0.69) and PC 2 (0.68), suggesting the secondary reaction had effects on both PC1 and PC 2 [38]. PC 3 (6.81%) and PC 4 (3.12%) had high loadings of Na^+^, Cl^−^, and Ca^2+^, suggesting sea salt and/or dust. Regarding FAMS, PC 1 (58.5%) had high loadings of all PAHs species, 2-NFR, and 1-NP, also suggesting the co-effects of combustion emission and secondary reaction, but the relative contribution was lower than that at WAMS. PC 2 (16.5%) had high loadings of Ca^2+^, Mg^2+^, and Cl^−^, suggesting the potential source was mainly dust. PC 3 (10.1%) had high loadings of K^+^ and NO_3_^−^, suggesting the combined impact of secondary reactions and biomass combustion. PC 4 (6.58%) and PC 5 (5.05%) had high loadings of NH_4_^+^, SO_4_^2−^, and 2-NP, suggesting secondary reactions. For Period 2, the potential emission sources of WSIIs could not be determined by PCA due to the limited number of samples. However, as indicated in Tables S1 and S2 (Supplementary Materials), Br^−^ was detected at WAMS and FAMS in Period 2, which is demonstrably enriched in fine sea-salt aerosol particles [49], suggesting a relatively large impact of sea salt on both WAMS and FAMS.

The high loading of all PAHs species at both WAMS and FAMS according to PCA could not adequately distinguish the emission sources of PAHs. Thus, the diagnostic ratios were used to further determine the emission sources. Figure S20 (Supplementary Materials) shows the ratios of [FR]/([FR] + [Pyr]) and [IDP]/([IDP] + [BgPe]) at WAMS and FAMS during the sampling periods. [Flu]/([Flu] + [Pyr]) and [IDP]/([IDP] + [BgPe]) correspond to the *X-* and *Y*-axes, respectively. According to the previous study, the source may be traffic emissions (including gasoline and diesel engines) if the ratio of [Flu]/([Flu] + [Pyr]) is between 0.2 and 0.5 [50] or between 0.6 and 0.7 [39], while it may be gasoline engine emissions of [IDP]/([IDP] + [BgPe]) between 0.25 and 0.65 [50]. As shown in Figure S20 (Supplementary Materials), despite the ratios on 16 April and 26 June not participating in the scatterplot due to the concentrations of IDP being below the LOD (Table S3, Supplementary Materials), all the ratios of [Flu]/([Flu] + [Pyr]) showed that PAHs were mainly emitted from traffic emissions, and most ratios of [IDP]/([IDP] + [BgPe]) originated from gasoline engine emissions. Therefore, it is surmised that the sources may have been traffic emissions from the Asian continent and ship engine emissions near the sites.

## 5. Conclusions

This is the first study on simultaneously collected daily PM_2.5_ samples and a comparison of their characteristics at WAMS and FAMS in the East Asian winter and summer monsoon periods. The results showed that the daily PM_2.5_ concentrations at FAMS were mostly higher than those at WAMS in the East Asian winter monsoon period, but the opposite trend was observed in the summer monsoon period. In addition to meteorological conditions imposing relatively large impacts on the target species, the diagnostic ratios showed that PAHs were mostly aged and NPAHs were mostly secondarily formed during the long-range transport process. The backward trajectories indicated that the air masses transported across a long distance arriving in Japan not only came from Asian continental coastal regions but also from more distant landlocked areas in the winter monsoon period, while, in the summer monsoon period, there was almost no air mass impact from the Asian continent at either FAMS or WAMS. However, the transport areas and daily air mass routes were different at WAMS and FAMS in both sampling periods. The acid–base balance results indicated that WSIIs at WAMS and FAMS in the summer monsoon period were more acidic than those in the winter monsoon period, and WSIIs at WAMS were more neutral than those at FAMS. PCA revealed that WSIIs at WAMS were mainly formed via the combustion process and secondary reactions, whereas those at FAMS originated from sea salt and dust. The ratios of [FR]/([FR] + [Pyr]) and [IDP]/([IDP] + [BgPe]) both indicated that PAHs mainly originated from traffic emissions at both sites.

Although it has been acknowledged that PAHs and NPAHs can easily react with several oxidant reagents, such as O_3_, NO_2_, and OH radicals in the atmosphere, the concentrations of PAHs and NPAHs were very low at these two background sites, and the concentrations of SO_2_, NO_2_, and O_3_ obtained from the online monitoring gaseous data (data not shown in this study) at these two background sites also indicated very low levels. Therefore, we did not focus on the artefacts of PAHs and NPAHs during the sampling periods in this study. Through this study, we obtained a better understanding of the similarities and differences between these two sites in the two typical periods which characterize these regions, and the findings also provide basic data for the establishment of prediction models of transboundary air pollutants in East Asia. The concentrations of each species in Period 2 were used as the baseline reference between two sites in the summer monsoon period. However, PM_2.5_ sampling for long periods is not possible due to the frequent typhoons in this period, and the fewer samples in Period 2 led to some uncertainty in the results. We will focus on improving this in future research.

## Figures and Tables

**Table 1:** Loading of PAHs, NPAHs, and WSIIs at WAMS and FAMS in the East Asian winter monsoon period according to principal component analysis. PC, principal component.

	WAMS				FAMS				
	PC 1	PC 2	PC 3	PC 4	PC 1	PC 2	PC 3	PC 4	PC 5
FR	**0.91**	0.26	−0.15	0.29	**0.73**	0.00	0.58	0.06	0.35
Pyr	**0.91**	0.31	−0.13	0.22	**0.75**	0.04	0.58	0.08	0.31
BaA	**0.87**	0.44	−0.11	0.02	**0.96**	0.14	0.14	0.07	0.15
Chr	**0.80**	0.54	−0.11	0.22	**0.85**	0.05	0.44	0.10	0.27
BbF	**0.83**	0.49	−0.09	0.22	**0.96**	0.16	0.21	−0.03	0.08
BkF	**0.83**	0.52	−0.11	0.14	**0.96**	0.17	0.18	0.02	0.08
BaP	**0.85**	0.50	−0.11	0.06	**0.96**	0.05	0.22	0.07	0.13
BgPe	**0.87**	0.47	−0.08	0.13	**0.95**	0.24	0.17	0.04	0.03
IDP	**0.85**	0.48	−0.10	0.18	**0.94**	0.16	0.22	0.02	0.02
2-NFR	0.69	0.68	0.02	0.12	**0.92**	−0.09	−0.16	0.24	0.10
2-NP	**0.77**	0.52	0.18	0.09	0.25	0.07	0.14	−0.19	**0.93**
1-NP	**0.74**	0.58	0.08	0.22	**0.84**	0.02	−0.11	0.25	0.15
Na^+^	−0.13	0.11	**0.95**	−0.07	0.58	0.54	0.06	0.18	0.56
NH_4_^+^	0.46	**0.86**	−0.14	0.07	0.16	−0.26	0.35	**0.88**	−0.06
K^+^	**0.77**	−0.09	0.12	0.47	0.09	0.27	**0.90**	0.13	0.14
Ca^2+^	0.32	0.16	0.13	**0.91**	−0.04	**0.87**	0.40	−0.21	−0.05
Mg^2+^	^−^ *				0.09	**0.87**	0.30	−0.03	0.33
Cl^−^	0.02	−0.28	**0.89**	0.26	0.35	**0.86**	−0.13	−0.27	−0.09
Br^−^	^−^ *				^−^ *				
NO_3_^−^	**0.83**	0.25	0.10	0.44	0.54	0.28	**0.74**	0.09	−0.06
SO_4_^2−^	0.36	**0.91**	−0.07	0.07	0.17	−0.11	−0.06	**0.96**	−0.09
% of Variance	75.1%	11.3%	6.81%	3.12%	58.5%	16.5%	10.1%	6.58%	5.05%

High factor loadings (>0.70) are marked in bold. * concentration below the limit of detection (LOD).

## Supplementary Materials

The following are available online at https://www.mdpi.com/1660-4601/17/21/8224/s1: Text S1. Quartz fiber filter treatment; Text S2. Sample pretreatment; Text S3. Quality control and quality assurance; Text S4. Calculated methods of cation equivalent (CE) and anion equivalent (AE); Text S5. Calculated methods of non-sea-salt WSIIs; Table S1. Abbreviation and limit of detection (LOD) of PAHs, NPAHs, and WSIIs; Table S2. Daily concentrations of PM_2.5_, and each individual PAH, NPAH, and WSII at WAMS in the two sampling periods; Table S3. Daily concentrations of PM_2.5_, and each individual PAH, NPAH, and WSII at FAMS in the two sampling periods; Table S4. Ratios of non-sea salt WSIIs factions at WAMS and FAMS during the East Asian winter monsoon period (Period 1); Figure S1. Percentage of each individual PAH at WAMS (a and c) and FAMS (b and d) in the East Asian winter monsoon period (Period 1, 10–20 april 2017) and summer monsoon period (Period 2, 25–29 June 2019); Figure S2. Percentage of individual NPAHs at WAMS a and FAMS b in the East Asian winter monsoon period (Period 1, 10–20 April 2017) and summer monsoon period (Period 2, 25–29 June 2019); Figure S3. Percentage of individual WSII and [WSIIs]/[PM_2.5_] at WAMS a and FAMS b in the East Asian winter monsoon period (Period 1, 10–20 April 2017) and summer monsoon period (Period 2, 25–29 June 2019); Figures S4–S14. Frequency analysis of backward trajectories at WAMS a and FAMS b on 10–20 April 2017. (☆) sampling sites at WAMS and FAMS; (■) longest air mass residence area; Figures S15–S19. Frequency analysis of backward trajectories at WAMS a and FAMS b on 25–29 June 2019. (☆) sampling sites at WAMS and FAMS; (■) longest air mass residence area; Figure S20. Ratios of [FR]/([FR] + [Pyr]) and [IDP]/([IDP] + [BgPe]) at WAMS and FAMS in the East Asian winter monsoon period (Period 1, 10–20 April 2017) and summer monsoon period (Period 2, 25–29 June 2019).
