# Supplementary material for: Comparative Analysis of PM2.5-Bound Polycyclic Aromatic Hydrocarbons (PAHs), Nitro-PAHs (NPAHs), and Water-Soluble Inorganic Ions (WSIIs) at Two Background Sites in Japan
Source: Int J Environ Res Public Health. 2020 Nov 6;17(21):8224. doi: 10.3390/ijerph17218224 (PMC7664402; doi:10.3390/ijerph17218224)
Supplement: Supplementary file 1 [file ijerph-17-08224-s001.pdf]

International Journal of *Environmental Research and Public Health*

MDPI

*Article*

# Comparative Analysis of $PM_{2.5}$-Bound Polycyclic Aromatic Hydrocarbons (PAHs), Nitro-PAHs (NPAHs), and Water-Soluble Inorganic Ions (WSIIs) at Two Background Sites in Japan

**Lu Yang [1], Lulu Zhang [1], Hao Zhang [1], Quanyu Zhou [1], Xuan Zhang [1], Wanli Xing [1], Akinori Takami [2], Kei Sato [2], Atsushi Shimizu [2], Ayako Yoshino [2], Naoki Kaneyasu [3], Atsushi Matsuki [4], Kazuichi Hayakawa [4], Akira Toriba [5], and Ning Tang [4, 5, *]**

[1] Graduate School of Medical Sciences, Kanazawa University, Kakuma-machi, Kanazawa 920-1192, Japan.
[2] National Institute for Environmental Studies, 16-2 Onogawa, Tsukuba, Ibaraki, 305-8506, Japan.
[3] National Institute of Advanced Industrial Science and Technology, 16-1 Onogawa, Tsukuba, Ibaraki, 305-8569, Japan.
[4] Institute of Nature and Environmental Technology, Kanazawa University, Kakuma-machi, Kanazawa 920-1192, Japan.
[5] Institute of Medical, Pharmaceutical and Health Sciences, Kanazawa University, Kakuma-machi, Kanazawa 920-1192, Japan.

* Correspondence: n_tang@staff.kanazawa-u.ac.jp; Tel.: +81-76-234-4455

**Supplementary Materials contents:**

**Text S1**. Quartz fiber Filter treatment**.**
**Text S2**. Sample pretreatment.
**Text S3**. Quality control and quality assurance.
**Text S4**. Calculated methods of cation equivalent (CE) and anion equivalent (AE).
**Text S5**. Calculated methods of non-sea-salt WSIIs.

**Table S1**. Abbreviation and limit of detection (LOD) of PAHs, NPAHs, and WSIIs.
**Table S2**. Daily concentrations of $PM_{2.5}$, each individual PAH, NPAH, and WSII at WAMS in two sampling periods.
**Table S3**. Daily concentrations of $PM_{2.5}$, each individual PAH, NPAH, and WSII at FAMS in two sampling periods.
**Table S4**. Ratios of non-sea salt WSIIs factions at WAMS and FAMS during the East Asian winter monsoon period (Period 1).

**Figure S1** Percentage of each individual PAH at WAMS ((a) and (c)) and FAMS ((b) and (d)) in the East Asian winter monsoon period (Period 1, 4/10 – 4/20, 2017) and summer monsoon period (Period 2, 6/25 – 6/29, 2019).
**Figure S2** Percentage of individual NPAHs at WAMS (a) and FAMS (b) in the East Asian winter monsoon period (Period 1, 4/10 – 4/20, 2017) and summer monsoon period (Period 2, 6/25 – 6/29, 2019).
**Figure S3** Percentage of individual WSII and [WSIIs]/[$PM_{2.5}$] at WAMS (a) and FAMS (b) in the East Asian winter monsoon period (Period 1, 4/10 – 4/20, 2017) and summer monsoon period (Period 2, 6/25 – 6/29, 2019).
**Figures S4 to S14** Frequency analysis of backward trajectories at WAMS (a) and FAMS (b) on April 10 to 20, 2017. (☆) means sampling sites at WAMS and FAMS, (■) means the longest of air mass residence area.

**Figures. S15 to S19** Frequency analysis of backward trajectories at WAMS (a) and FAMS (b) on June 25 to 29, 2019. (☆) means sampling sites at WAMS and FAMS, (■) means the longest of air mass residence area.

**Figure S20** Ratios of [FR]/([FR]+[Pyr]) and [IDP]/([IDP]+[BgPe]) at WAMS and FAMS in the East Asian winter monsoon period (Period 1, 4/10 – 4/20, 2017) and summer monsoon period (Period 2, 6/25 – 6/29, 2019).

**Text S1**. Quartz fiber Filter treatment

To ensure the accuracy of the calculation of $PM_{2.5}$ concentration, quartz fiber filters were put into the desiccator with constant room temperature (21.5 ± 1.5 °C) and humidity (50 ± 5%) for 48 h. Before sampling, each filter was weighed and take the average of twice, then packaged by aluminum foil. After sampling, the filters were put into the desiccator under the same condition as before sampling, also weighed and take the average of twice. After weighing, the filters were packaged by aluminum foil and kept sealed in plastic bags and stored at -25 °C until analyzed. The concentration of each $PM_{2.5}$ sample was calculated as: $PM_{2.5}$ concentration = (weigh $_{after}$ – weigh $_{before}$)/ total volume.

**Text S2**. Sample pretreatment

For PAHs and NPAHs, one-third of each $PM_{2.5}$ filter (about 130 $cm^2$) was cut into small pieces and placed in flasks. PAH and NPAH internal standards (pyrene-$d_{10}$ (Pyr-$d_{10}$), benzo[*a*]pyrene-$d_{12}$, (BaP-$d_{12}$, and 2-fluoro-7-nitrofluorene (FNF)) were added to the filter. Then, benzene: ethanol (3: 1, v/v) was added for extracting. After ultrasonic extraction twice, the solution was filtered, and the extracts were washed successively with 5% sodium hydroxide solution, 20% sulfuric acid solution, and distilled water. The solution was concentrated with a rotary evaporator to 100 μL after adding dimethyl sulfoxide. Then, adding ethanol to make the residue up to 1 mL. Finally, the solution was filtered into a vial by a 0.45 μm membrane filter (HLC-DISK13, Kanto Chemical CO., Inc., Tokyo, Japan). After pretreatment, the analysis solution was injected into an HPLC and the target PAHs and NPAHs were determined by the fluorescence detection system.

For WSIIs, one-third of each $PM_{2.5}$ filter (about 130 $cm^2$) was cut into small pieces and placed in polypropylene tubes. Ultrapure water was added to each tube and put at room temperature for 10 min. After ultrasonic extraction for 30 min, the solution was transferred through a 0.45 μm filter (SLHPX13NK, Merck Millipore Ltd. Tullagreen, Carrigtwohill, Co. Cork IRELAND) filtered into two portions. After pretreatment, the analysis solution was injected into an ion chromatography system to separately detect anions and cations.

**Text S3**. Quality control and quality assurance

To ensure the accuracy of chemicals detection, quartz fiber filters were roasted at 600 °C for 4 h. To checking the background contamination during transportation, blank filters were also analyzed, and no target chemicals were determined, indicating there was no contamination during the transport. To check the HPLC and IC analysis methods, PAHs, NPAHs, and WSIIs standard solution had injected into the systems before actual samples detecting, respectively. The calibration curves of all PAHs, NPAHs, and WSIIs had good linearity (r > 0.998). Pyr-$d_{10}$ was used to quantify the 4-ring (FR, Pyr, BaA, and Chr) PAHs, BaP-$d_{12}$ was used to quantify the 5- (BbF, BkF, and BaP) and 6-ring (BgPe and IDP) PAHs and FNF was used to quantify the three NPAHs (1-, 2-NPs and 2-NFR). The recoveries of internal standards of PAHs and NPAHs ranged from 80% to 95% in this study. The limit of determination (LOD) of each PAH, NPAH, and WSII are shown in **Table S1**.

**Text S4**. Calculated methods of cation equivalent (CE) and anion equivalent (AE)

The value of AE and CE were calculated as followed:

$$AE = [SO_4^{2-}]/48 + [NO_3^-]/62 + [Cl^-]/35.5 + [Br^-]/79.9 \quad \text{(Equation 1)}$$

$$CE = [NH_4^+]/18 + [Mg^{2+}]/12.2 + [Ca^{2+}]/20 + [K^+]/39 + [Na^+]/23 \quad \text{(Equation 2)}$$

$[SO_4^{2-}]$, $[NO_3^-]$, $[Cl^-]$, $[Br^-]$, $[NH_4^+]$, $[Mg^{2+}]$, $[Ca^{2+}]$, $[K^+]$, and $[Na^+]$ are the concentrations.

**Text S5**. Calculated methods of non-sea-salt WSIIs.

The value of non-sea-salt (nss-) WSIIs were calculated as followed:

$[nss\text{-} SO_4^{2-}] = [SO_4^{2-}] - [Na^+] \times 0.2516$ (Equation 3)

$[nss\text{-} Ca^{2+}] = [Ca^{2+}] - [Na^+] \times 0.038$ (Equation 4)

$[nss\text{-}K^+] = [K^+] - [Na^+] \times 0.037$ (Equation 5)

$[SO_4^{2-}]$, $[Ca^{2+}]$, $[K^+]$, and $[Na^+]$ are the concentrations.

**Table S1.** Abbreviation and limit of detection (LOD) of PAHs, NPAHs, and WSIIs.

| Species | Abbreviation | LOD [a] |
|---|---|---|
| Fluoranthene | FR | 16.2 |
| Pyrene | Pyr | 30.3 |
| Benz[*a*]anthracene | BaA | 9.1 |
| Chrysene | Chr | 34.3 |
| Benzo[*b*]fluoranthene | BbF | 55.5 |
| Benzo[*k*]fluoranthene | BkF | 8.8 |
| Benzo[*a*]pyrene | BaP | 8.8 |
| Benzo[*ghi*]perylene | BgPe | 55.3 |
| Indeno[1,2,3-*cd*]pyrene | IDP | 82.9 |
| Total PAHs | ΣPAHs | |
| 1-Nitropyrene | 1-NP | 12.4 |
| 2-Nitropyrene | 2-NP | 6.2 |
| 2-Nitrofluoranthene | 2-NFR | 5.0 |
| Total NPAHs | ΣNPAHs | |
| Sodium | $Na^+$ | 0.28 |
| Ammonium | $NH_4^+$ | 0.14 |
| Potassium | $K^+$ | 0.43 |
| Calcium | $Ca^{2+}$ | 0.13 |
| Magnesium | $Mg^{2+}$ | 0.30 |
| Chloride | $Cl^-$ | 0.23 |
| Sulfate | $SO_4^{2-}$ | 0.02 |
| Nitrate | $NO_3^-$ | 0.08 |
| Bromine | $Br^-$ | 0.03 |
| Total WSIIs | ΣWSIIs | |

[a]: Unit of pg/mL for PAHs and NPAHs, μg/mL for WSIIs.

**Table S2.** Daily concentrations of $PM_{2.5}$, each individual PAH, NPAH, and WSII at WAMS in two sampling periods.

| | East Asian winter monsoon period (Period 1) | | | | | | | | | | | East Asian summer monsoon period (Period 2) | | | | |
|---|---|---|---|---|---|---|---|---|---|---|---|---|---|---|---|---|
| | 4/10 | 4/11 | 4/12 | 4/13 | 4/14 | 4/15 | 4/16 | 4/17 | 4/18 | 4/19 | 4/20 | 6/25 | 6/26 | 6/27 | 6/28 | 6/29 |
| $PM_{2.5}$ (μg/m$^3$) | 6.90 | 2.33 | 4.14 | 17.2 | 21.2 | 6.60 | 10.9 | 7.99 | 4.50 | 5.82 | 7.26 | 9.43 | 12.4 | 6.30 | 5.84 | 5.47 |
| PAHs (pg/m$^3$) | | | | | | | | | | | | | | | | |
| FR | 111 | 57.3 | 120 | 143 | 238 | 111 | 115 | 44.3 | 38.0 | 78.6 | 95.0 | 6.45 | 38.4 | 13.5 | 6.29 | 9.23 |
| Pyr | 62.9 | 35.2 | 70.5 | 81.0 | 147 | 70.8 | 73.0 | 26.7 | 22.3 | 52.4 | 57.6 | 20.3 | 42.1 | 23.9 | 19.0 | 21.5 |
| BaA | 11.4 | 6.06 | 11.4 | 13.0 | 26.1 | 18.0 | 14.9 | 5.05 | 4.74 | 11.6 | 12.2 | 1.49 | 9.29 | 2.42 | 0.89 | 2.04 |
| Chr | 31.9 | 17.6 | 33.8 | 45.8 | 88.2 | 45.2 | 55.1 | 17.3 | 13.7 | 24.5 | 28.0 | 2.13 | 20.9 | 6.08 | 0.81 | 1.57 |
| BbF | 43.5 | 22.8 | 37.6 | 51.3 | 103 | 51.1 | 56.7 | 22.9 | 18.0 | 28.5 | 32.5 | 2.78 | 32.4 | 8.84 | 2.12 | 5.41 |
| BkF | 15.4 | 8.02 | 13.1 | 16.4 | 35.5 | 19.5 | 20.4 | 7.62 | 6.24 | 10.4 | 12.4 | 0.68 | 11.3 | 2.86 | 0.39 | 1.37 |
| BaP | 23.2 | 12.1 | 18.5 | 23.1 | 53.0 | 30.8 | 29.5 | 11.9 | 9.21 | 17.2 | 22.1 | 1.53 | 19.2 | 4.40 | 0.89 | 2.61 |
| BgPe | 48.8 | 26.6 | 42.4 | 53.7 | 116 | 62.4 | 61.7 | 25.5 | 22.3 | 37.7 | 44.5 | 3.35 | 39.4 | 7.87 | 2.23 | 4.69 |
| IDP | 22.9 | 12.6 | 18.2 | 25.4 | 49.8 | 26.4 | 27.7 | 12.9 | 9.74 | 16.3 | 19.3 | 2.12 | 28.3 | 5.95 | 1.56 | 3.36 |
| NPAHs (pg/m$^3$) | | | | | | | | | | | | | | | | |
| 2-NFR | 3.89 | 1.41 | 1.12 | 3.47 | 10.1 | 4.29 | 6.24 | 2.28 | 1.16 | 2.65 | 1.33 | 0.71 | 1.04 | 0.14 | 0.07 | 0.15 |
| 2-NP | 0.21 | 0.07 | 0.11 | 0.25 | 0.67 | 0.25 | 0.33 | 0.16 | 0.08 | 0.33 | 0.12 | 0.04 | 0.15 | 0.04 | 0.02 | 0.03 |
| 1-NP | 0.48 | 0.26 | 0.54 | 1.00 | 2.13 | 0.87 | 1.20 | 0.46 | 0.23 | 0.84 | 0.32 | 0.12 | 0.19 | **0.02** | **0.02** | **0.02** |
| WSIIs (μg/m$^3$) | | | | | | | | | | | | | | | | |
| $Na^+$ | 0.083 | **0.008** | 0.091 | 0.116 | 0.132 | 0.160 | 0.106 | 0.156 | 0.301 | 0.233 | 0.079 | **0.006** | **0.006** | **0.006** | **0.006** | 0.065 |
| $NH_4^+$ | 0.554 | 0.429 | 0.435 | 0.744 | 1.862 | 1.294 | 1.826 | 0.612 | 0.407 | 0.293 | 0.535 | 0.639 | 1.043 | 0.157 | 0.270 | 0.111 |
| $K^+$ | 0.131 | **0.012** | 0.150 | 0.158 | 0.180 | 0.089 | 0.095 | 0.068 | 0.087 | 0.082 | 0.113 | **0.009** | **0.009** | **0.009** | **0.009** | **0.009** |
| $Ca^{2+}$ | 0.054 | **0.003** | 0.063 | 0.219 | 0.141 | 0.054 | 0.046 | 0.086 | 0.047 | 0.026 | **0.003** | **0.003** | **0.003** | **0.003** | **0.003** | **0.003** |
| $Mg^{2+}$ | **0.008** | **0.008** | **0.008** | **0.008** | **0.008** | **0.008** | **0.008** | **0.008** | **0.008** | **0.008** | **0.008** | **0.006** | **0.006** | **0.006** | **0.006** | **0.006** |
| $Cl^-$ | 0.051 | **0.006** | 0.059 | 0.068 | 0.085 | 0.006 | 0.006 | 0.094 | 0.141 | 0.087 | 0.039 | **0.005** | **0.005** | **0.005** | **0.005** | **0.005** |
| $Br^-$ | **0.001** | **0.001** | **0.001** | **0.001** | **0.001** | **0.001** | **0.001** | **0.001** | **0.001** | **0.001** | **0.001** | 0.009 | 0.013 | 0.004 | 0.004 | **0.001** |
| $NO_3^-$ | 0.206 | 0.056 | 0.214 | 0.454 | 0.827 | 0.120 | 0.221 | 0.069 | 0.095 | 0.172 | 0.104 | 0.042 | 0.029 | **0.002** | **0.002** | 0.015 |
| $SO_4^{2-}$ | 1.418 | 1.195 | 1.278 | 2.193 | 4.825 | 3.898 | 5.151 | 2.040 | 1.689 | 1.074 | 1.510 | 2.054 | 2.992 | 0.512 | 0.772 | 0.476 |

Bold font means concentrations calculated by half of LOD to represent species which below LOD.

**Table S3.** Daily concentrations of $PM_{2.5}$, each individual PAH, NPAH, and WSII at FAMS in two sampling periods.

| 0 | East Asian winter monsoon period (Period 1) | | | | | | | | | | | East Asian summer monsoon period (Period 2) | | | | |
|---|---|---|---|---|---|---|---|---|---|---|---|---|---|---|---|---|
| | 4/10 | 4/11 | 4/12 | 4/13 | 4/14 | 4/15 | 4/16 | 4/17 | 4/18 | 4/19 | 4/20 | 6/25 | 6/26 | 6/27 | 6/28 | 6/29 |
| $PM_{2.5}$ (μg/m$^3$) | 20.1 | 16.7 | 23.6 | 19.1 | 14.9 | 15.8 | 12.0 | 8.90 | 27.9 | 78.5 | 17.5 | 7.51 | 2.79 | 5.39 | 5.49 | 3.61 |
| PAHs (pg/m$^3$) | | | | | | | | | | | | | | | | |
| FR | 68.5 | 226 | 216 | 158 | 44.8 | 9.74 | 7.01 | 40.5 | 143 | 98.8 | 63.9 | 35.6 | 5.55 | 6.24 | 5.99 | 6.42 |
| Pyr | 42.7 | 133 | 119 | 87.4 | 25.0 | 4.66 | 2.63 | 21.5 | 89.8 | 60.7 | 38.8 | 39.2 | 19.5 | 19.9 | 20.5 | 20.2 |
| BaA | 21.7 | 24.9 | 20.1 | 13.7 | 5.07 | 3.37 | 3.44 | 8.08 | 20.8 | 12.5 | 8.01 | 4.04 | 0.55 | 1.01 | 1.23 | 1.48 |
| Chr | 37.2 | 73.7 | 65.4 | 49.3 | 13.2 | 3.33 | 1.24 | 14.3 | 55.1 | 31.9 | 21.8 | 18.9 | 0.86 | 0.67 | 2.22 | 2.12 |
| BbF | 94.1 | 105 | 83.9 | 70.3 | 23.0 | 10.8 | 4.50 | 29.6 | 80.5 | 61.7 | 48.3 | 24.0 | 0.86 | 2.09 | 3.09 | 2.77 |
| BkF | 31.2 | 34.3 | 27.2 | 22.6 | 7.35 | 3.48 | 0.98 | 10.1 | 28.5 | 19.9 | 14.7 | 6.87 | 0.37 | 0.58 | 0.89 | 0.68 |
| BaP | 35.3 | 45.8 | 34.9 | 27.1 | 8.19 | 2.33 | 1.38 | 12.6 | 35.2 | 18.5 | 15.2 | 8.44 | **0.05** | 0.53 | 1.09 | 1.52 |
| BgPe | 111 | 118 | 92.1 | 82.4 | 25.5 | 13.9 | 6.33 | 44.3 | 111 | 79.6 | 55.6 | 16.3 | 0.79 | 1.97 | 3.03 | 3.33 |
| IDP | 44.7 | 51.2 | 39.3 | 35.7 | 11.3 | 6.49 | **0.07** | 18.2 | 44.7 | 31.1 | 29.7 | 13.1 | **0.08** | 1.23 | 1.40 | 2.11 |
| NPAHs (pg/m$^3$) | | | | | | | | | | | | | | | | |
| 2-NFR | 9.92 | 8.61 | 6.62 | 6.00 | 1.49 | 0.34 | 0.14 | 5.94 | 9.07 | 0.89 | 1.00 | 0.63 | 0.08 | 0.10 | 0.21 | 0.23 |
| 2-NP | 0.70 | 0.84 | 10.6 | 0.96 | 0.11 | 0.03 | 0.02 | 0.36 | 0.64 | 1.25 | 0.17 | 0.11 | 0.06 | 0.06 | 0.06 | 0.09 |
| 1-NP | 2.08 | 1.51 | 1.50 | 1.51 | 1.43 | 0.12 | **0.02** | 0.54 | 1.74 | 0.55 | 0.40 | 0.13 | **0.02** | **0.02** | **0.02** | **0.02** |
| WSIIs (μg/m$^3$) | | | | | | | | | | | | | | | | |
| $Na^+$ | 0.159 | 0.156 | 0.234 | 0.087 | 0.114 | 0.091 | 0.061 | 0.105 | 0.199 | 0.178 | 0.061 | **0.007** | 0.044 | **0.006** | 0.054 | 0.118 |
| $NH_4^+$ | 0.510 | 1.705 | 0.870 | 1.433 | 1.209 | 1.557 | 0.604 | 1.082 | 1.877 | 0.521 | 0.651 | 1.013 | 0.223 | 0.746 | 1.102 | 0.635 |
| $K^+$ | 0.012 | 0.164 | 0.140 | 0.148 | 0.109 | 0.091 | 0.081 | **0.012** | 0.134 | 0.171 | 0.096 | **0.010** | **0.009** | **0.009** | **0.009** | **0.009** |
| $Ca^{2+}$ | **0.004** | 0.080 | 0.254 | 0.211 | 0.141 | **0.004** | **0.004** | **0.004** | 0.335 | 1.378 | 0.215 | **0.003** | **0.003** | **0.003** | **0.003** | **0.003** |
| $Mg^{2+}$ | **0.008** | **0.008** | 0.056 | **0.008** | **0.008** | **0.008** | **0.008** | **0.008** | 0.056 | 0.093 | **0.008** | **0.007** | **0.006** | **0.006** | **0.006** | **0.006** |
| $Cl^-$ | 0.206 | 0.042 | 0.069 | **0.006** | **0.006** | **0.006** | **0.006** | **0.006** | 0.079 | 0.283 | **0.006** | **0.005** | **0.005** | **0.005** | **0.005** | **0.005** |
| $Br^-$ | **0.001** | **0.001** | **0.001** | **0.001** | **0.001** | **0.001** | **0.001** | **0.001** | **0.001** | **0.001** | **0.001** | 0.011 | 0.004 | 0.009 | 0.011 | 0.007 |
| $NO_3^-$ | 0.089 | 1.563 | 0.663 | 0.931 | 0.055 | 0.020 | 0.020 | 0.280 | 0.900 | 1.201 | 0.165 | **0.002** | **0.002** | **0.002** | **0.002** | 0.026 |
| $SO_4^{2-}$ | 3.008 | 3.505 | 2.492 | 3.442 | 3.508 | 4.483 | 1.811 | 3.019 | 4.755 | 1.981 | 2.222 | 2.930 | 0.783 | 2.467 | 3.616 | 2.174 |

Bold font means concentrations calculated by half of LOD to represent species which below LOD.

**Table S4.** Ratios of non-sea salt WSIIs factions at WAMS and FAMS during the East Asian winter monsoon period (Period 1).

| | WAMS | | | FAMS | | |
|---|---|---|---|---|---|---|
| | [nss-$SO_4^{2-}$]/[$SO_4^{2-}$] | [nss-$Ca^{2+}$]/[$Ca^{2+}$] | [nss-$K^+$]/[$K^+$] | [nss-$SO_4^{2-}$]/[$SO_4^{2-}$] | [nss-$Ca^{2+}$]/[$Ca^{2+}$] | [nss-$K^+$]/[$K^+$] |
| 4/10 | 0.99 | 0.94 | 0.98 | 0.99 | - [b] | 0.50 |
| 4/11 | - [a] | - [a] | - [a] | 0.99 | 0.92 | 0.96 |
| 4/12 | 0.98 | 0.94 | 0.98 | 0.98 | 0.96 | 0.94 |
| 4/13 | 0.99 | 0.98 | 0.97 | 0.99 | 0.98 | 0.98 |
| 4/14 | 0.99 | 0.96 | 0.97 | 0.99 | 0.97 | 0.96 |
| 4/15 | 0.99 | 0.88 | 0.93 | 0.99 | - [b] | 0.96 |
| 4/16 | 0.99 | 0.91 | 0.96 | 0.99 | - [b] | 0.97 |
| 4/17 | 0.98 | 0.93 | 0.92 | 0.99 | - [b] | 0.67 |
| 4/18 | 0.96 | 0.75 | 0.87 | 0.99 | 0.98 | 0.95 |
| 4/19 | 0.95 | 0.66 | 0.89 | 0.98 | 1.00 | 0.96 |
| 4/20 | 0.99 | 0.12 | 0.97 | 0.99 | 0.99 | 0.98 |

[a]: $Na^+$ below the LOD; [b]: $Ca^{2+}$ below the LOD.

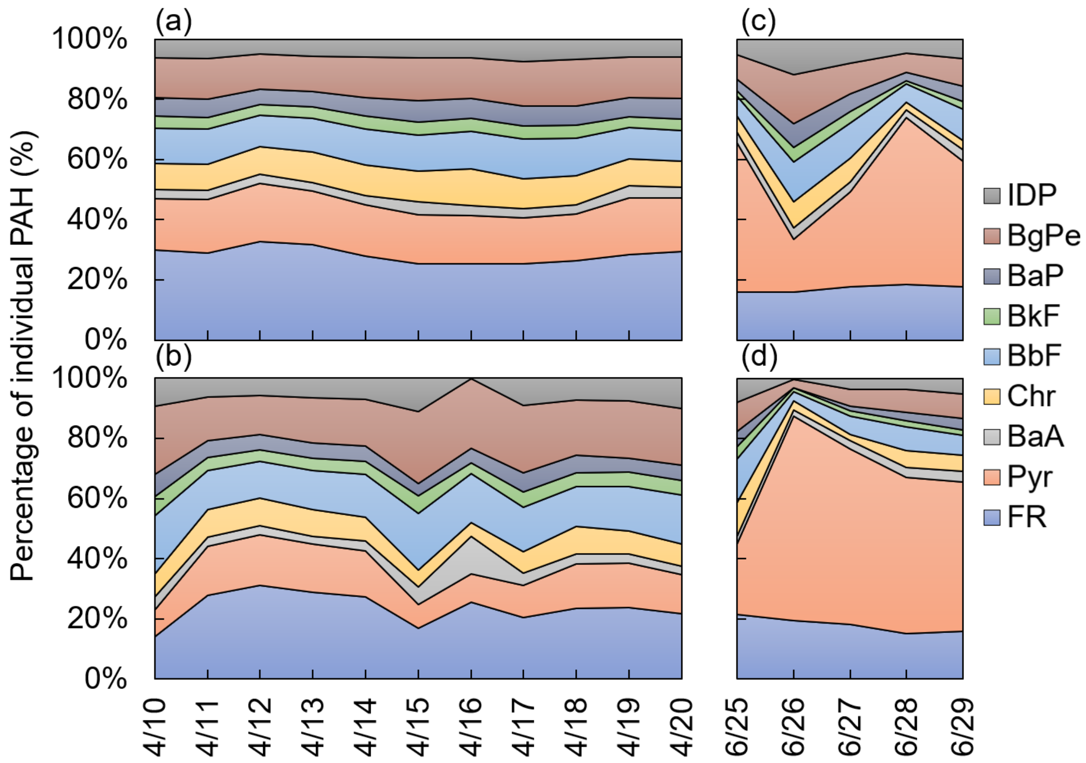


**Figure S1.** Percentage of each individual PAH at WAMS ((a) and (c)) and FAMS ((b) and (d)) in the East Asian winter monsoon period (Period 1, 4/10 – 4/20, 2017) and summer monsoon period (Period 2, 6/25 – 6/29, 2019).

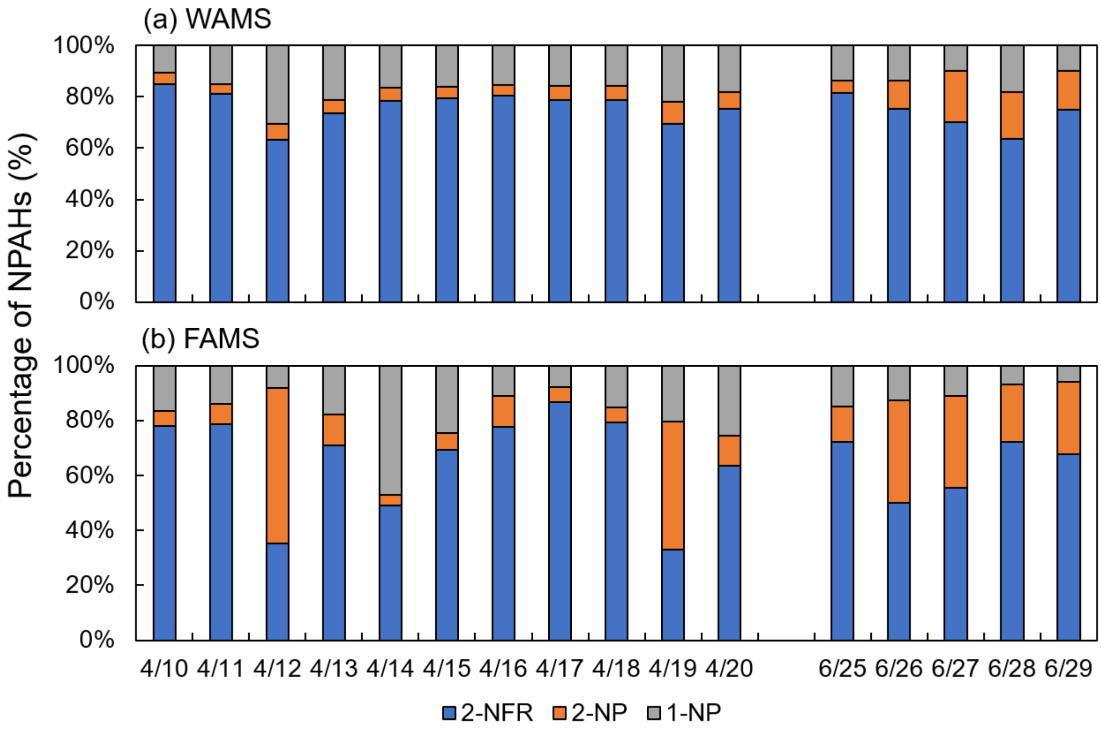


**Figure S2.** Percentage of individual NPAHs at WAMS (a) and FAMS (b) in the East Asian winter monsoon period (Period 1, 4/10 – 4/20, 2017) and summer monsoon period (Period 2, 6/25 – 6/29, 2019).

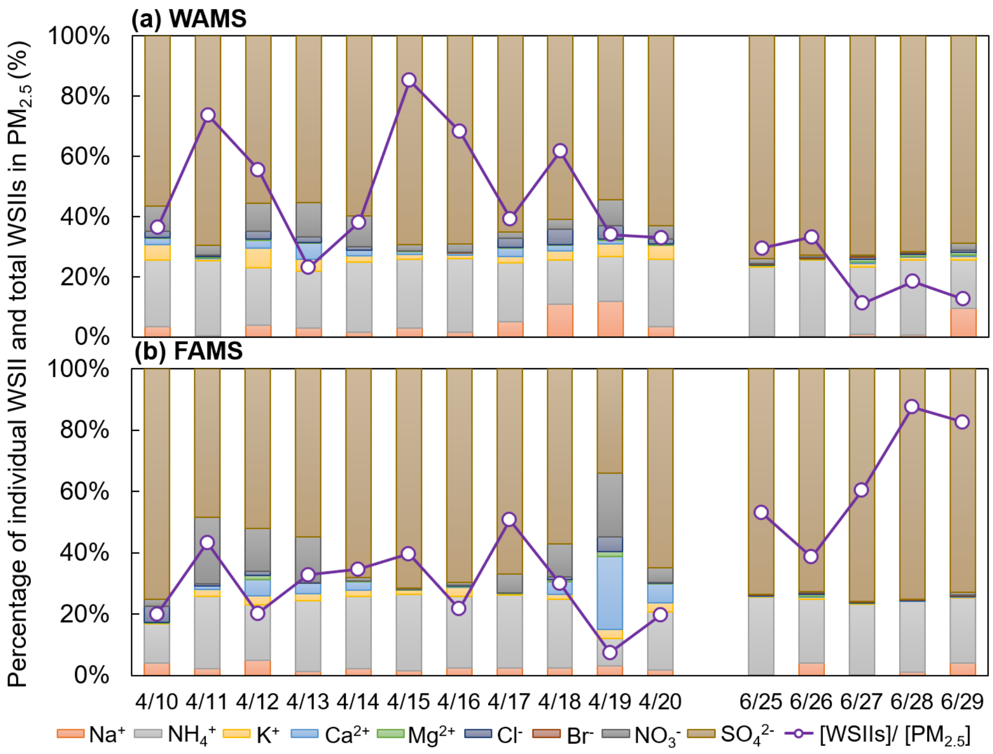


**Figure S3.** Percentage of individual WSII and [WSIIs]/[$PM_{2.5}$] at WAMS (a) and FAMS (b) in the East Asian winter monsoon period (Period 1, 4/10 – 4/20, 2017) and summer monsoon period (Period 2, 6/25 – 6/29, 2019).

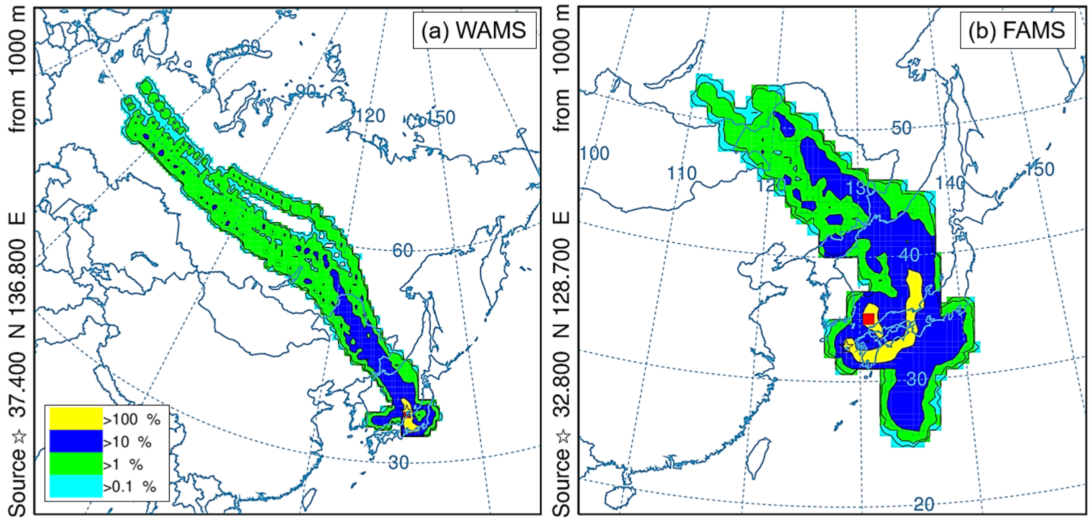


**Figure S4.** Frequency analysis of backward trajectories at WAMS (a) and FAMS (b) on April 10, 2017. (☆) means sampling sites at WAMS and FAMS, (■) means the longest of air mass residence area.

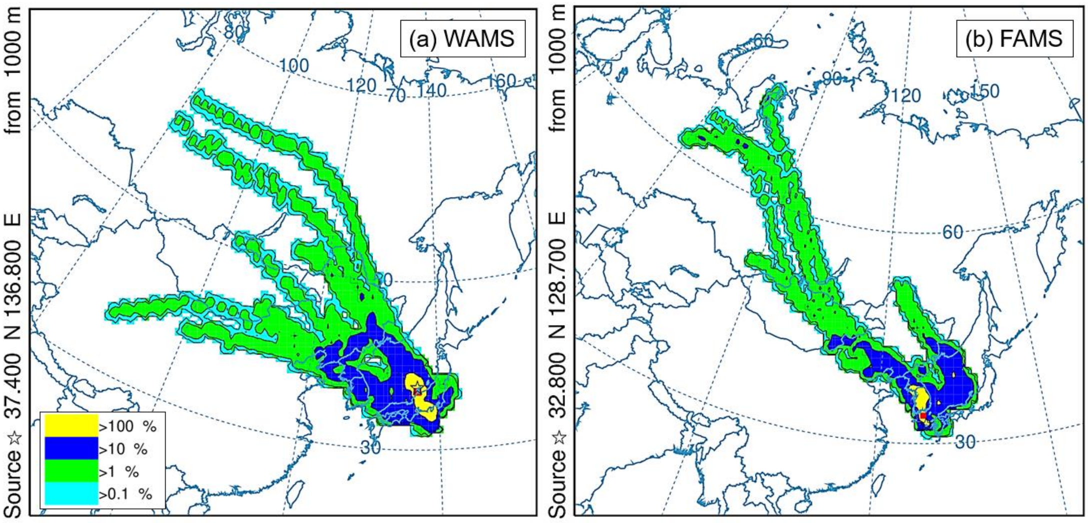


**Figure S5**. Frequency analysis of backward trajectories at WAMS (a) and FAMS (b) on April 11, 2017. (☆) means sampling sites at WAMS and FAMS, (■) means the longest of air mass residence area.

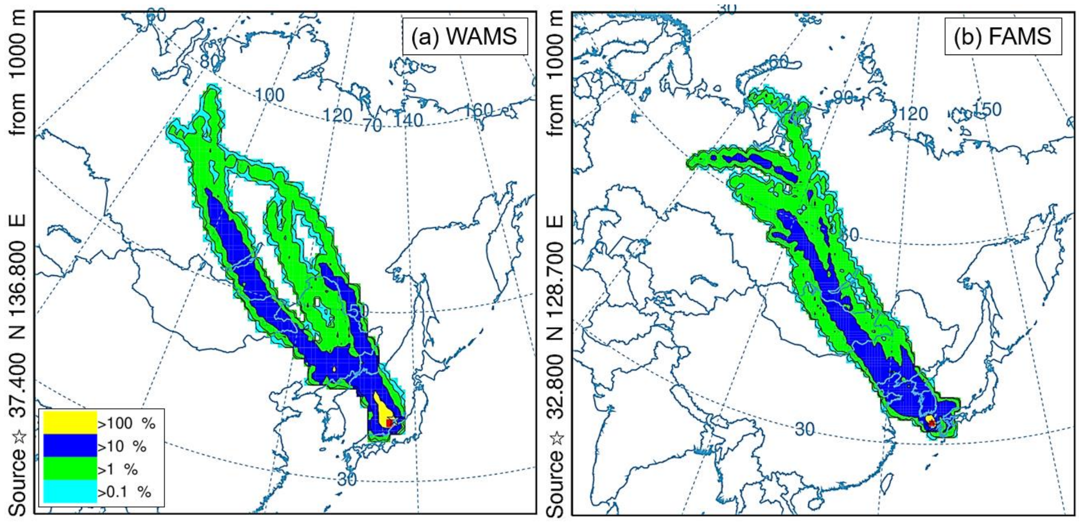


**Figure S6.** Frequency analysis of backward trajectories at WAMS (a) and FAMS (b) on April 12, 2017. (☆) means sampling sites at WAMS and FAMS, (■) means the longest of air mass residence area.

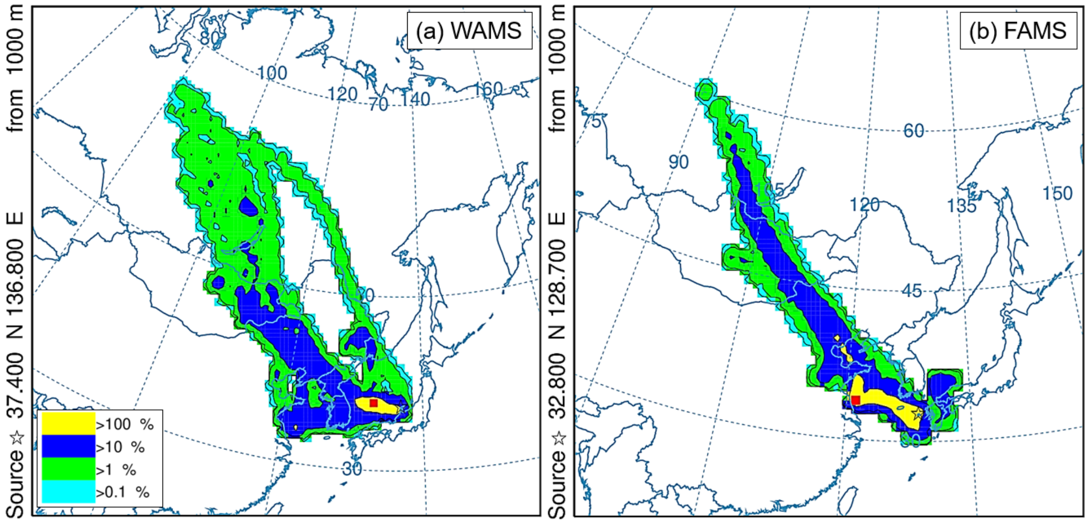


**Figure S7.** Frequency analysis of backward trajectories at WAMS (a) and FAMS (b) on April 13, 2017. (☆) means sampling sites at WAMS and FAMS, (■) means the longest of air mass residence area.

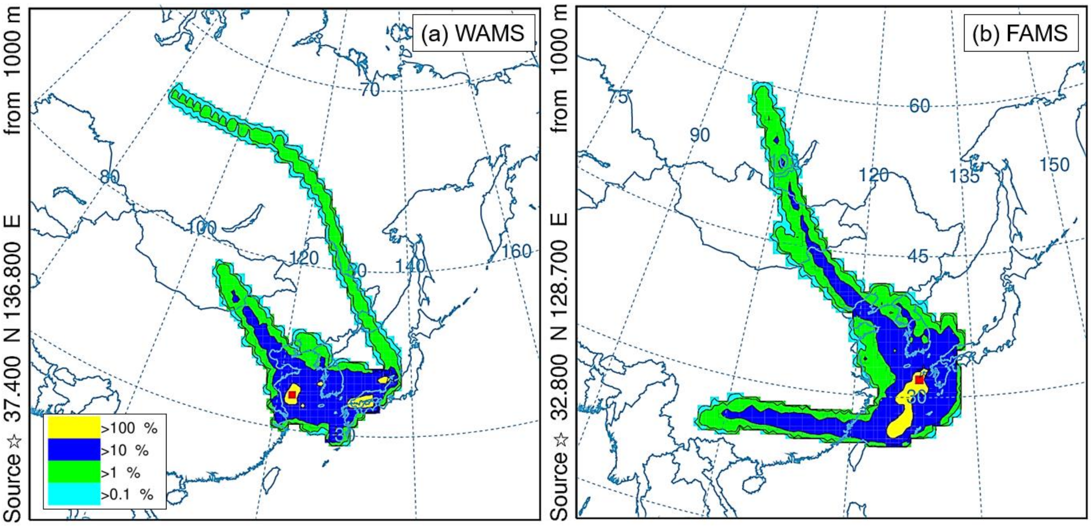


**Figure S8.** Frequency analysis of backward trajectories at WAMS (a) and FAMS (b) on April 14, 2017. (☆) means sampling sites at WAMS and FAMS, (■) means the longest of air mass residence area.

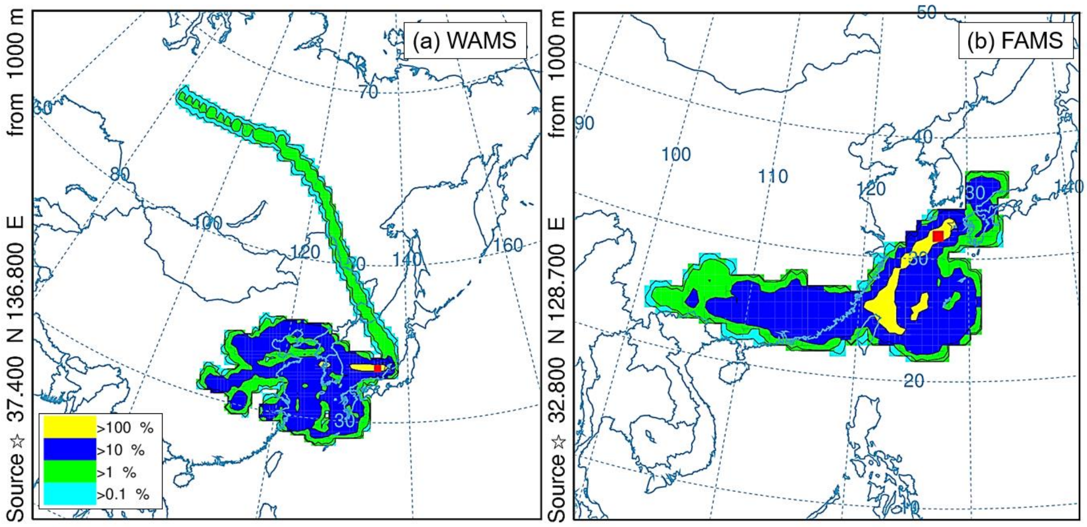


**Figure S9.** Frequency analysis of backward trajectories at WAMS (a) and FAMS (b) on April 15, 2017. (☆) means sampling sites at WAMS and FAMS, (■) means the longest of air mass residence area.

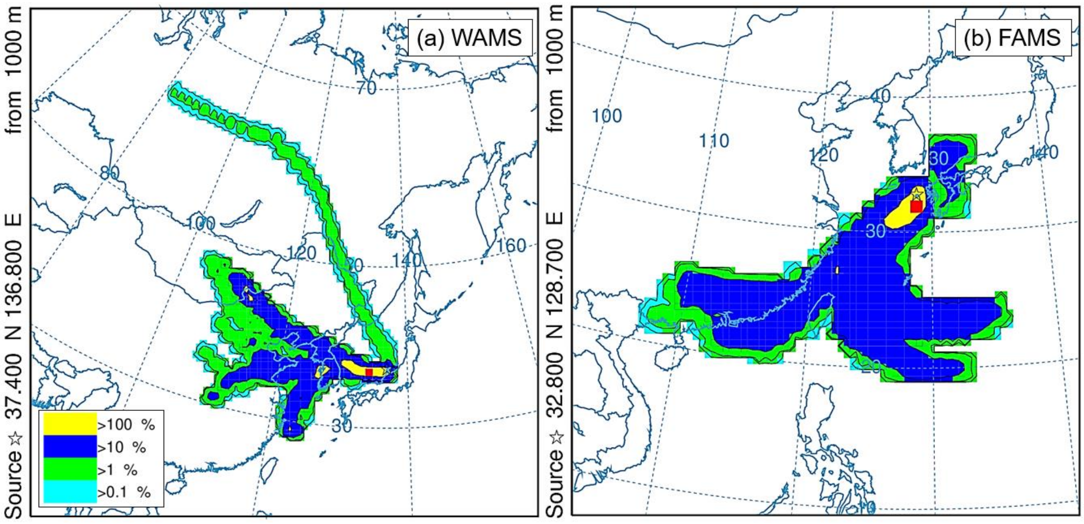

**Figure S10.** Frequency analysis of backward trajectories at WAMS (a) and FAMS (b) on April 16, 2017. (☆) means sampling sites at WAMS and FAMS, (■) means the longest of air mass residence area.

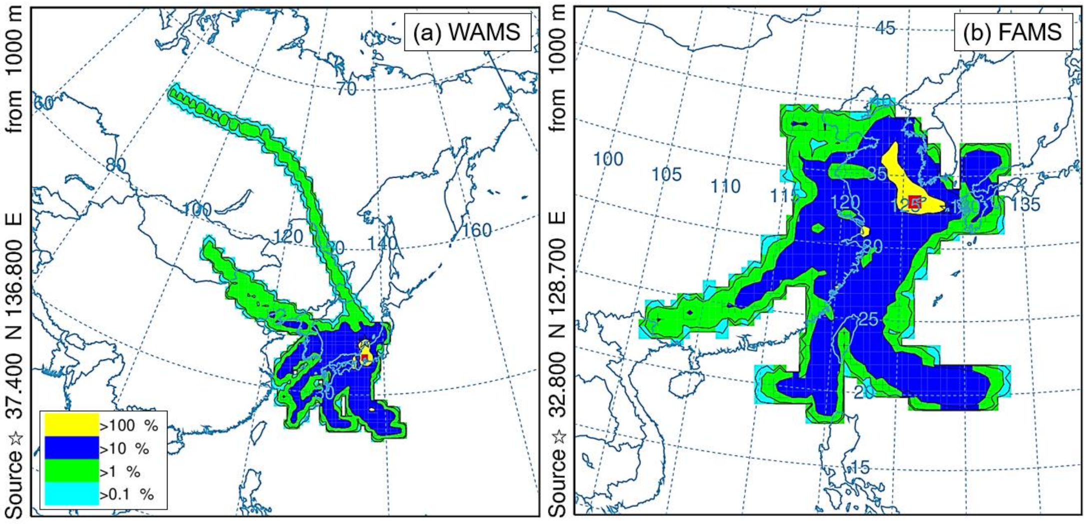


**Figure S11.** Frequency analysis of backward trajectories at WAMS (a) and FAMS (b) on April 17, 2017. (☆) means sampling sites at WAMS and FAMS, (■) means the longest of air mass residence area.

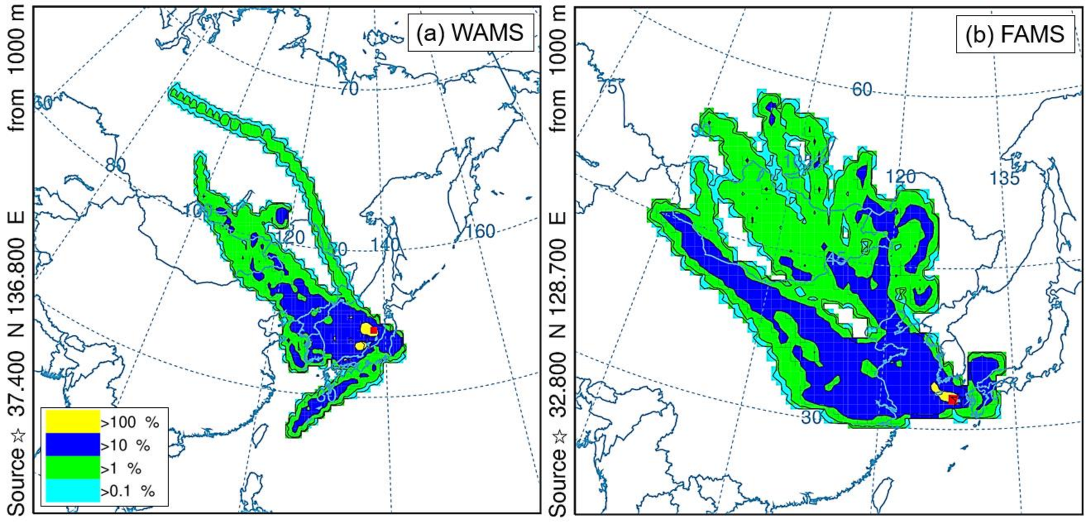


**Figure S12.** Frequency analysis of backward trajectories at WAMS (a) and FAMS (b) on April 18, 2017. (☆) means sampling sites at WAMS and FAMS, (■) means the longest of air mass residence area.

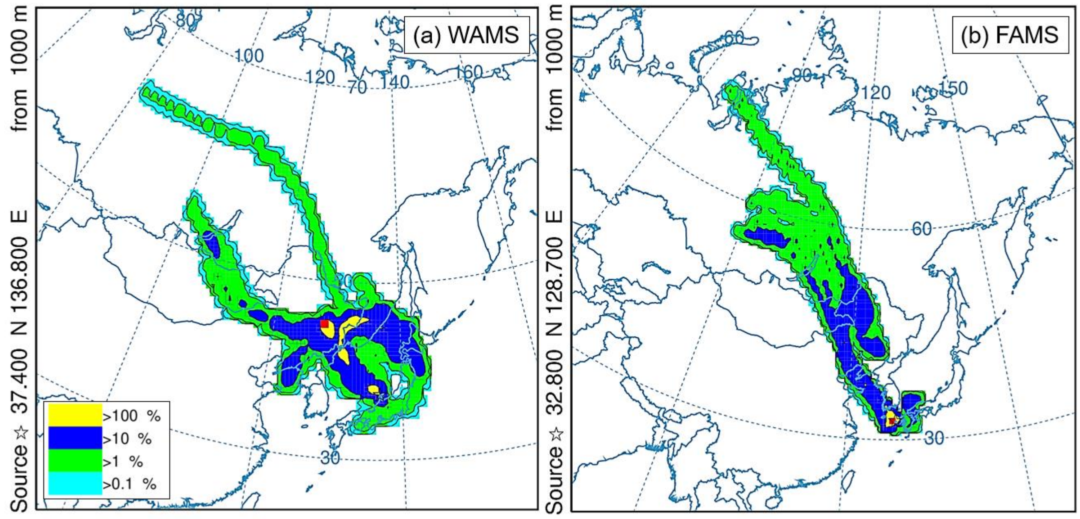

**Figure S13.** Frequency analysis of backward trajectories at WAMS (a) and FAMS (b) on April 19, 2017. (☆) means sampling sites at WAMS and FAMS, (■) means the longest of air mass residence area.

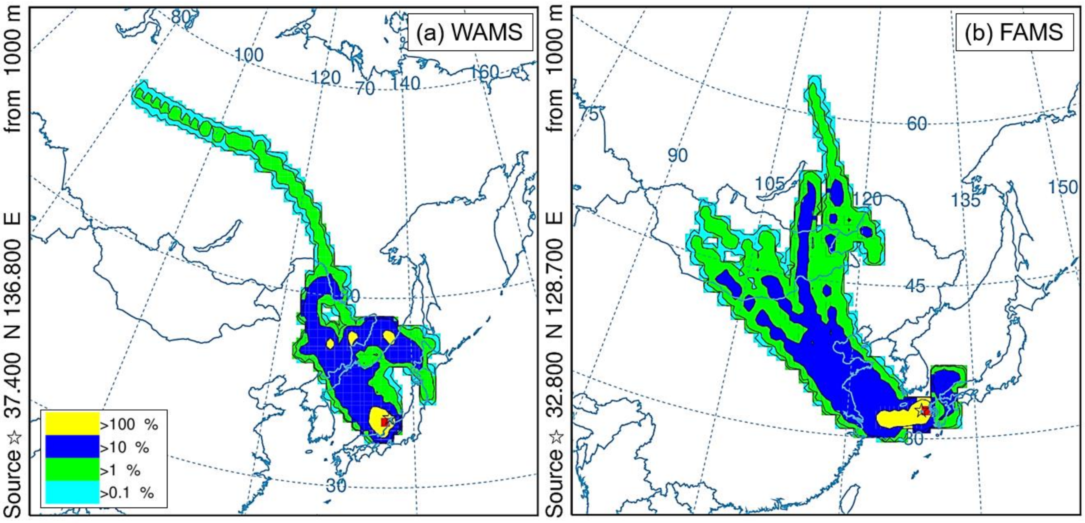


**Figure S14.** Frequency analysis of backward trajectories at WAMS (a) and FAMS (b) on April 20, 2017. (☆) means sampling sites at WAMS and FAMS, (■) means the longest of air mass residence area.

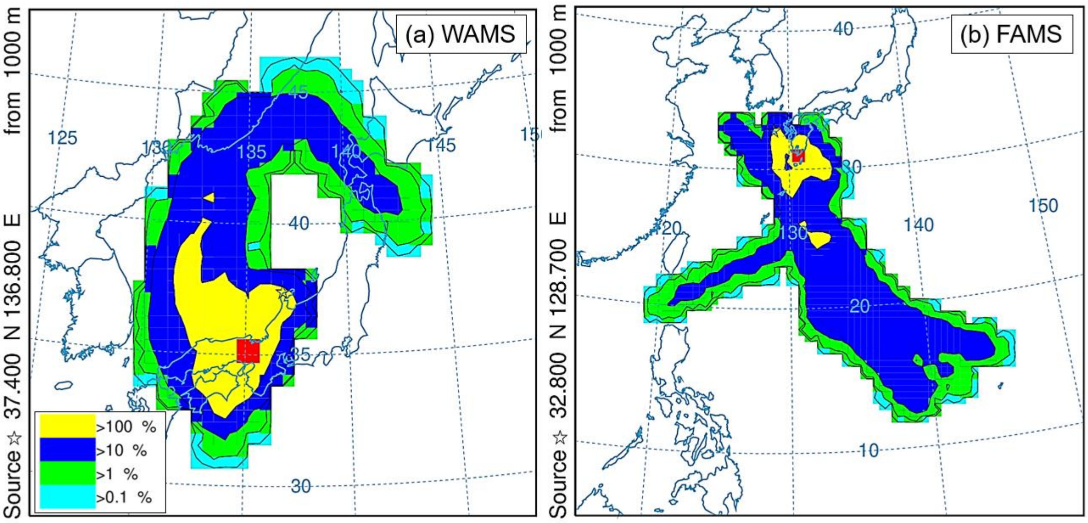


**Figure S15.** Frequency analysis of backward trajectories at WAMS (a) and FAMS (b) on June 25, 2019. (☆) means sampling sites at WAMS and FAMS, (■) means the longest of air mass residence area.

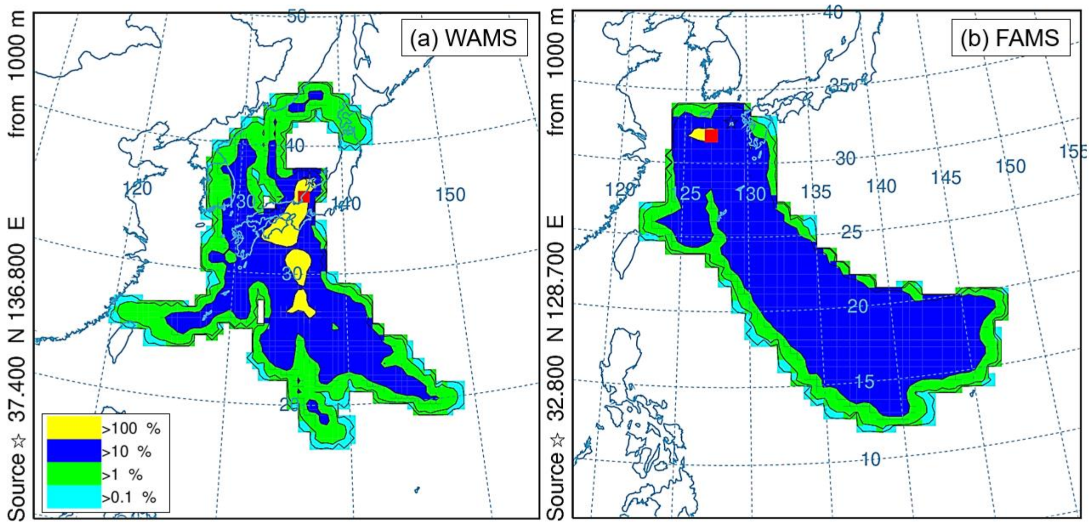

**Figure S16.** Frequency analysis of backward trajectories at WAMS (a) and FAMS (b) on June 26, 2019. (☆) means sampling sites at WAMS and FAMS, (■) means the longest of air mass residence area.

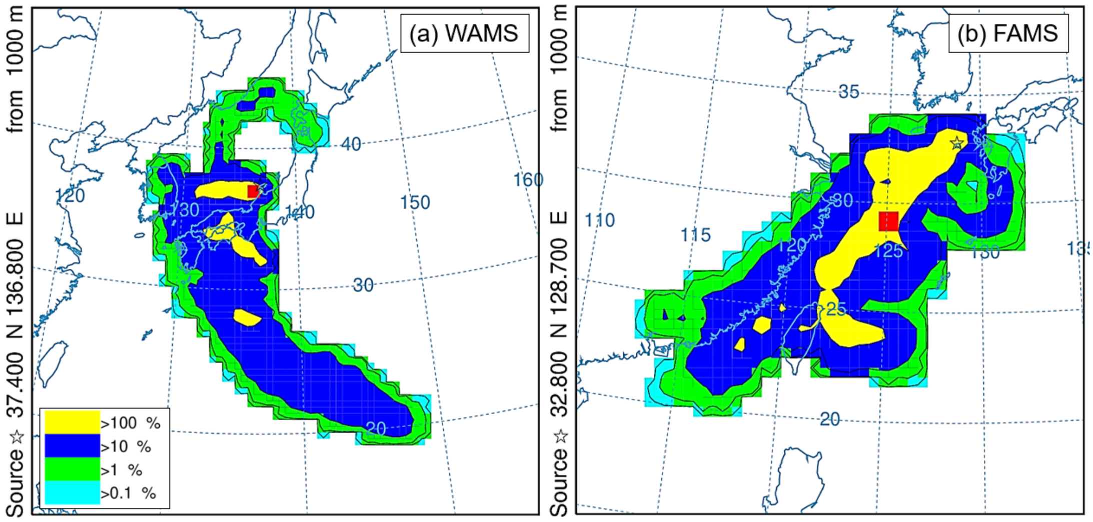


**Figure S17.** Frequency analysis of backward trajectories at WAMS (a) and FAMS (b) on June 27, 2019. (☆) means sampling sites at WAMS and FAMS, (■) means the longest of air mass residence area.

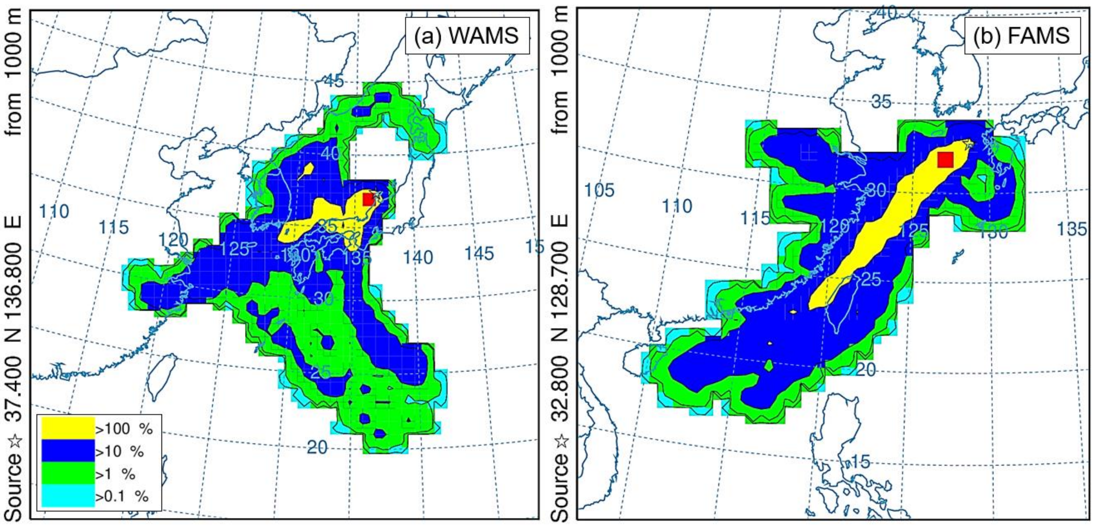


**Figure S18.** Frequency analysis of backward trajectories at WAMS (a) and FAMS (b) on June 28, 2019. (☆) means sampling sites at WAMS and FAMS, (■) means the longest of air mass residence area.

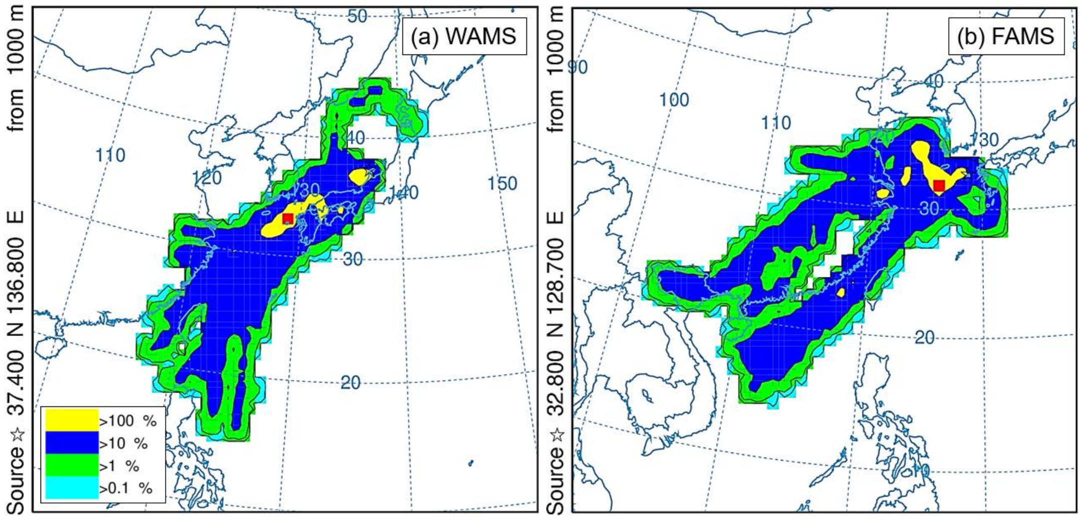

141 **Figure S19.** Frequency analysis of backward trajectories at WAMS (a) and FAMS (b) on June 29, 2019.
142 (☆) means sampling sites at WAMS and FAMS, (■) means the longest of air mass residence area.

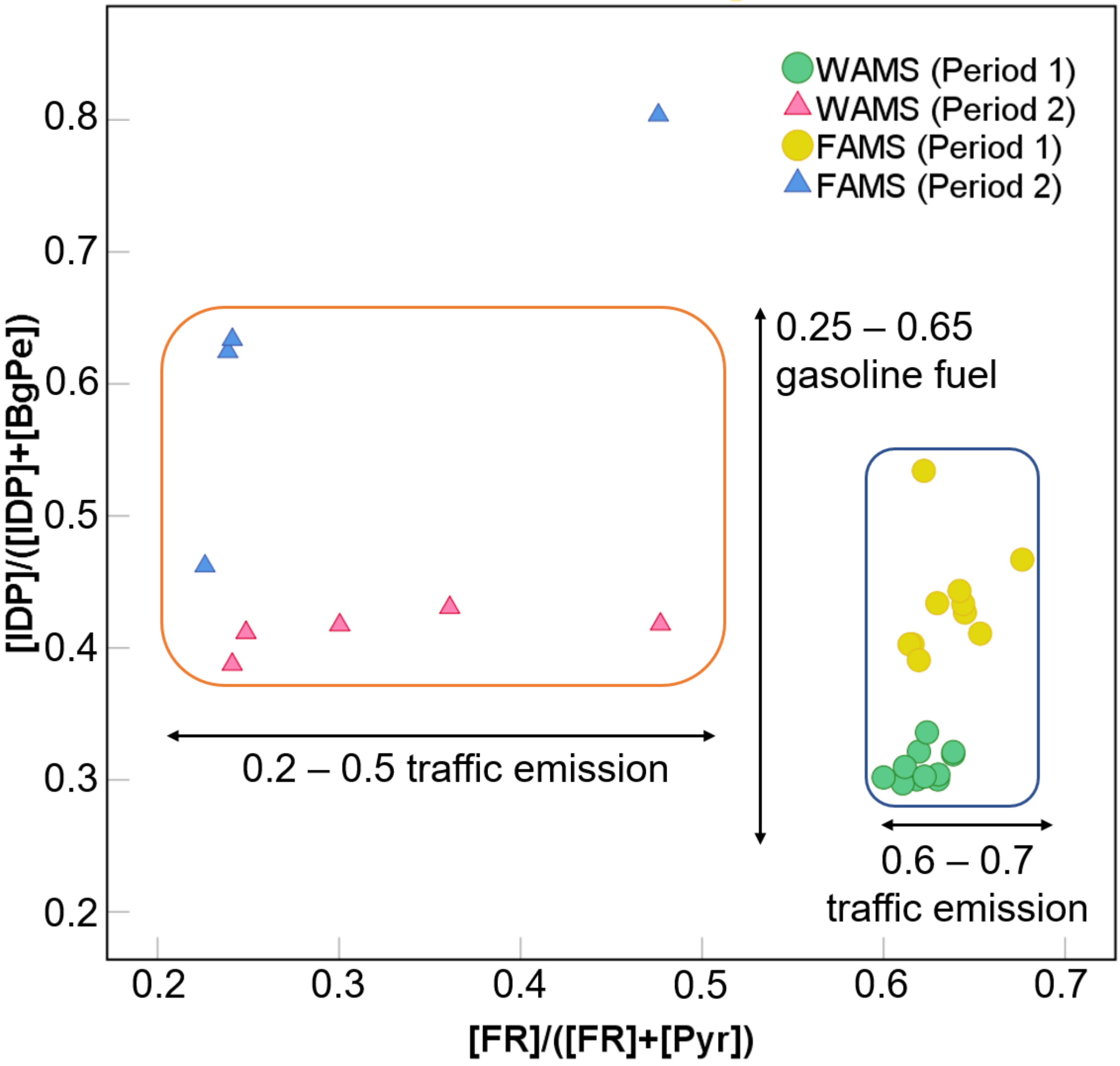


143 **Figure S20.** Ratios of [FR]/([FR]+[Pyr]) and [IDP]/([IDP]+[BgPe]) at WAMS and FAMS in the East
144 Asian winter monsoon period (Period 1, 4/10 – 4/20, 2017) and summer monsoon period (Period 2,
145 6/25 – 6/29, 2019).
